# The Rubella Virus Capsid Is an Anti-Apoptotic Protein that Attenuates the Pore-Forming Ability of Bax

**DOI:** 10.1371/journal.ppat.1001291

**Published:** 2011-02-17

**Authors:** Carolina S. Ilkow, Ing Swie Goping, Tom C. Hobman

**Affiliations:** 1 Department of Cell Biology, University of Alberta, Edmonton, Canada; 2 School of Molecular and Systems Medicine, University of Alberta, Edmonton, Canada; 3 Department of Biochemistry, University of Alberta, Edmonton, Canada; 4 Department of Oncology, University of Alberta, Edmonton, Canada; 5 Department of Medical Microbiology and Immunology, University of Alberta, Edmonton, Canada; 6 Li Ka Shing Institute of Virology, University of Alberta, Edmonton, Canada; University of Alabama at Birmingham, United States of America

## Abstract

Apoptosis is an important mechanism by which virus-infected cells are eliminated from the host. Accordingly, many viruses have evolved strategies to prevent or delay apoptosis in order to provide a window of opportunity in which virus replication, assembly and egress can take place. Interfering with apoptosis may also be important for establishment and/or maintenance of persistent infections. Whereas large DNA viruses have the luxury of encoding accessory proteins whose primary function is to undermine programmed cell death pathways, it is generally thought that most RNA viruses do not encode these types of proteins. Here we report that the multifunctional capsid protein of Rubella virus is a potent inhibitor of apoptosis. The main mechanism of action was specific for Bax as capsid bound Bax and prevented Bax-induced apoptosis but did not bind Bak nor inhibit Bak-induced apoptosis. Intriguingly, interaction with capsid protein resulted in activation of Bax in the absence of apoptotic stimuli, however, release of cytochrome *c* from mitochondria and concomitant activation of caspase 3 did not occur. Accordingly, we propose that binding of capsid to Bax induces the formation of hetero-oligomers that are incompetent for pore formation. Importantly, data from reverse genetic studies are consistent with a scenario in which the anti-apoptotic activity of capsid protein is important for virus replication. If so, this would be among the first demonstrations showing that blocking apoptosis is important for replication of an RNA virus. Finally, it is tempting to speculate that other slowly replicating RNA viruses employ similar mechanisms to avoid killing infected cells.

## Author Summary

Among the variety of defense systems employed by mammalian cells to combat virus infection, apoptosis or programmed cell death is the most drastic response. Some large DNA viruses encode proteins whose sole function is to block apoptosis. Conversely, very little is known about whether RNA viruses encode analogous proteins. In many cases, RNA viruses are able to replicate before cell death occurs, which may be one reason why so little thought has been given to this topic. However, a number of RNA viruses, some of which are important human pathogens, have slow replication cycles and it stands to reason that they must block apoptosis during this time period. Here we show that the multifunctional capsid protein of Rubella virus is a potent inhibitor of apoptosis. Data from reverse genetic experiments suggest that the anti-apoptotic function of a virus-encoded protein is important for replication of an RNA virus. We anticipate that other slowly replicating RNA viruses may employ similar mechanisms and, as such, these studies have implications for development of novel anti-virals and vaccines.

## Introduction

Rubella virus (RV) is an enveloped positive strand RNA virus in the family *Togaviridae* and is the sole member of the genus Rubivirus (reviewed in [1]). Humans are the only natural host for RV and in most cases the virus causes a systemic infection the symptoms of which include maculopapular rash, lymphadenopathy, low-grade fever, conjunctivitis and sore throat. However, RV infections can be complicated by the appearance of acute or chronic arthralgia, arthritis, thrombocytopenia and encephalopathy. *In utero* infection during the first trimester of pregnancy often results in a characteristic series of birth defects known as congenital Rubella syndrome. Worldwide, RV is thought to cause more birth defects that any other infectious agent yet, very little is known about molecular aspects of viral pathogenesis. A number of studies suggest that viral persistence may underlie some of the most serious aspects of infection including congenital Rubella syndrome and arthritis [2], [3], [4], [5], [6].

Among the togavirus family, RV is unique in that its replication is associated with mitochondria. The link between RV infection and this organelle first became apparent when analysis of purified virions revealed that cardiolipin, a phospholipid that is only found in mitochondria, is a significant component of the RV envelope [7]. Subsequently, it was discovered that RV infected cells exhibit striking mitochondrial defects. Virus infection induces clustering of mitochondria in the perinuclear region as well as formation of electron-dense plaques between apposing mitochondrial cisternae: structures that have been termed confronting membranes [8], [9]. The function of these structures is not known but expression of capsid protein in the absence of other RV proteins is sufficient to induce their formation [10]. A large pool of the capsid protein localizes to the surface of mitochondria [11] and the inter-mitochondrial plaques [12] but given that assembly of RV virions occurs primarily on Golgi membranes, the targeting of the capsid to this organelle likely reflects a nonstructural function of this protein.

The studies described above underscore the close link between the capsid protein and mitochondria in RV biology and form the basis for our central hypothesis; that association of the RV capsid protein with mitochondria is important for virus replication. All viruses must contend with host cell anti-viral mechanisms and large DNA viruses have the luxury of harboring in many cases, multiple genes devoted to thwarting host cell defenses (reviewed in [13]). In contrast, simple RNA viruses express a very limited number of proteins, most of which are directly involved in replication and virus assembly. Accordingly, it is beneficial if not essential that these viral proteins have multiple functions.

It is well documented that togavirus infection often results in apoptotic death of mammalian cells (reviewed in [14], [15]) and to our knowledge, there are no published studies showing that members of this virus family inhibit programmed cell death pathways. With the exception of RV, togavirus replication and virus egress from vertebrate cells occurs within 4–6 hours followed by extensive death by apoptosis within 24 hours. Accordingly, for most togaviruses, preventing apoptosis is likely not required in order for efficient replication to occur. In contrast, the replication cycle for RV is unusually slow; the eclipse period is at least 12 hours and viral titers peak virion secretion occurs between 48–72 (reviewed in [16]). RV-induced apoptosis in mammalian cells has been reported but generally, extensive cytopathic effect is not a hallmark of RV infection. When apoptosis does occur, it is not until 5–7 days post-infection that maximum levels are reached [17] and this is well past the peak virus production phase. In the present study, we report that the capsid protein blocks apoptosis in RV infected cells most likely to allow sufficient time for virus replication. This process occurs at the level of mitochondria through a Bax-dependent pathway.

## Results

### Cells infected with RV are resistant to apoptosis

**Figure 1:**
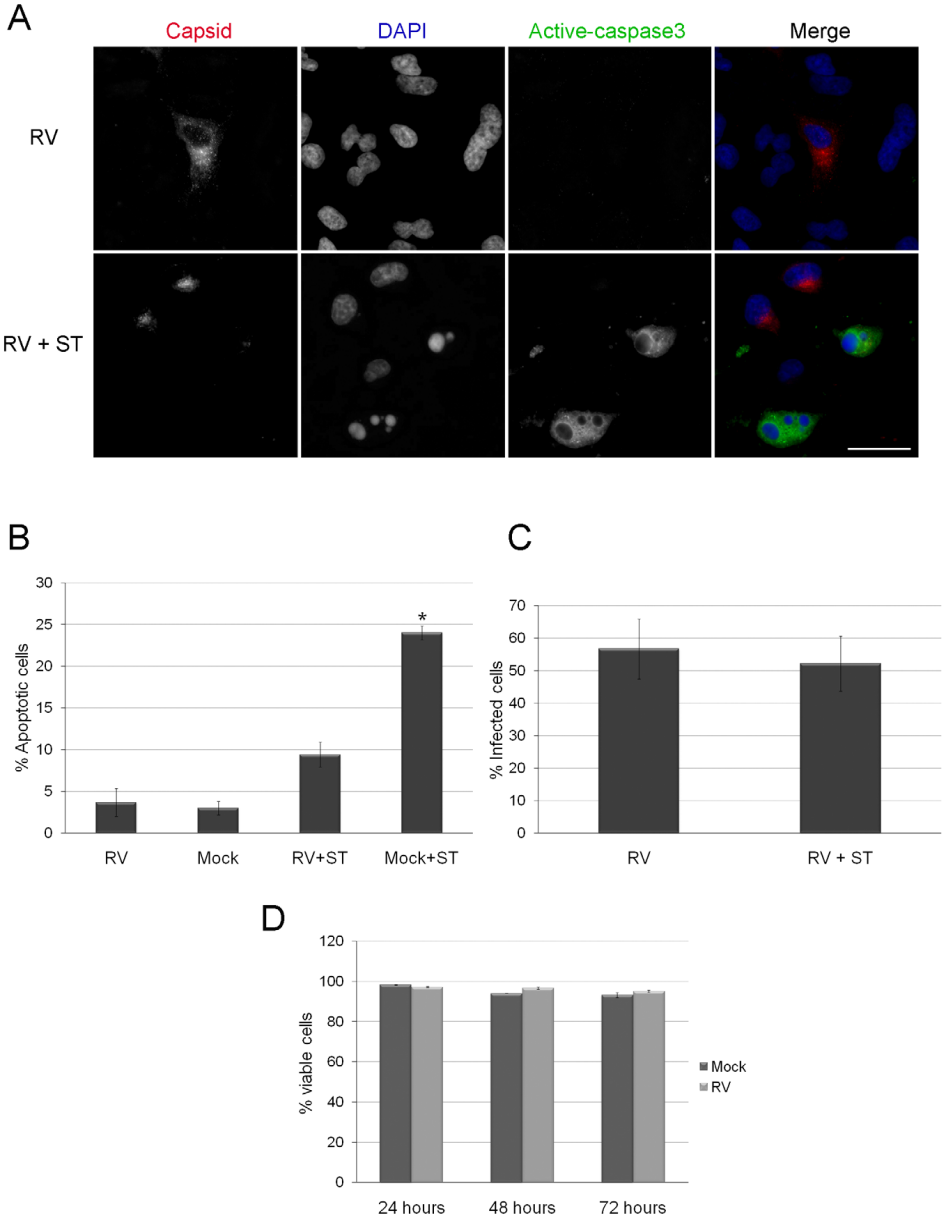
Caspase 3 activation is blocked in RV infected cells. A. A549 were infected with RV (MOI = 1) for 42 hours after which they were treated with staurosporine (ST) for 6 hours. Samples were then processed for indirect immunofluorescence using rabbit anti-caspase 3 and mouse anti-capsid. Primary antibodies were detected with donkey anti-rabbit Alexa488 and chicken anti-mouse Alexa594. Nuclei were counter stained with DAPI. Scale bar  = 20 µm. B. The percentages of cells among mock and RV infected cells treated with ST expressing active caspase 3 were determined and plotted. Student's *t*-Test was performed to determine statistical significance. *p* = ≤0.001. Percentages were determined from three independent experiments in which at least 100 cells for each experiment were scored. C. To demonstrate that ST treatment did not result in selective loss of infected cells, the percentages of RV infected cells were determined in control (RV) and ST-treated (RV + ST) samples. D. The percentage of viable cells in mock and RV infected samples were determined by Trypan blue dye exclusion at the indicated time points. Error bars indicate standard deviations calculated from three independent experiments.

We reasoned that in order for RV replication and virion secretion to increase through 48 hours and beyond, programmed cell death must be inhibited during this period. Accordingly, we compared the levels of apoptosis in RV and mock-infected A549 cells by indirect immunofluorescence using an antibody specific for activated caspase 3. Interestingly, less than 5% of infected cells exhibited signs of apoptosis 48 hours post-infection (Figure 1A and B). Moreover, when challenged with the kinase inhibitor staurosporine, a potent inducer of apoptosis [18], RV infected cells were significantly more resistant to apoptosis than mock infected cells. Specifically, the percentage of caspase 3-positive cells was almost three fold lower in the infected samples. This was not due to detachment of infected cells as data in Figure 1C show that treatment with staurosporine did not cause significant loss of infected cells. Finally, Figure 1D shows that even after 72 hours, RV infection does not significantly affect the percentage of viable A549 cells. Together, these data indicate that RV-infected A549 cells are resistant to programmed cell death.

### Expression of capsid protein inhibits apoptosis

**Figure 2:**
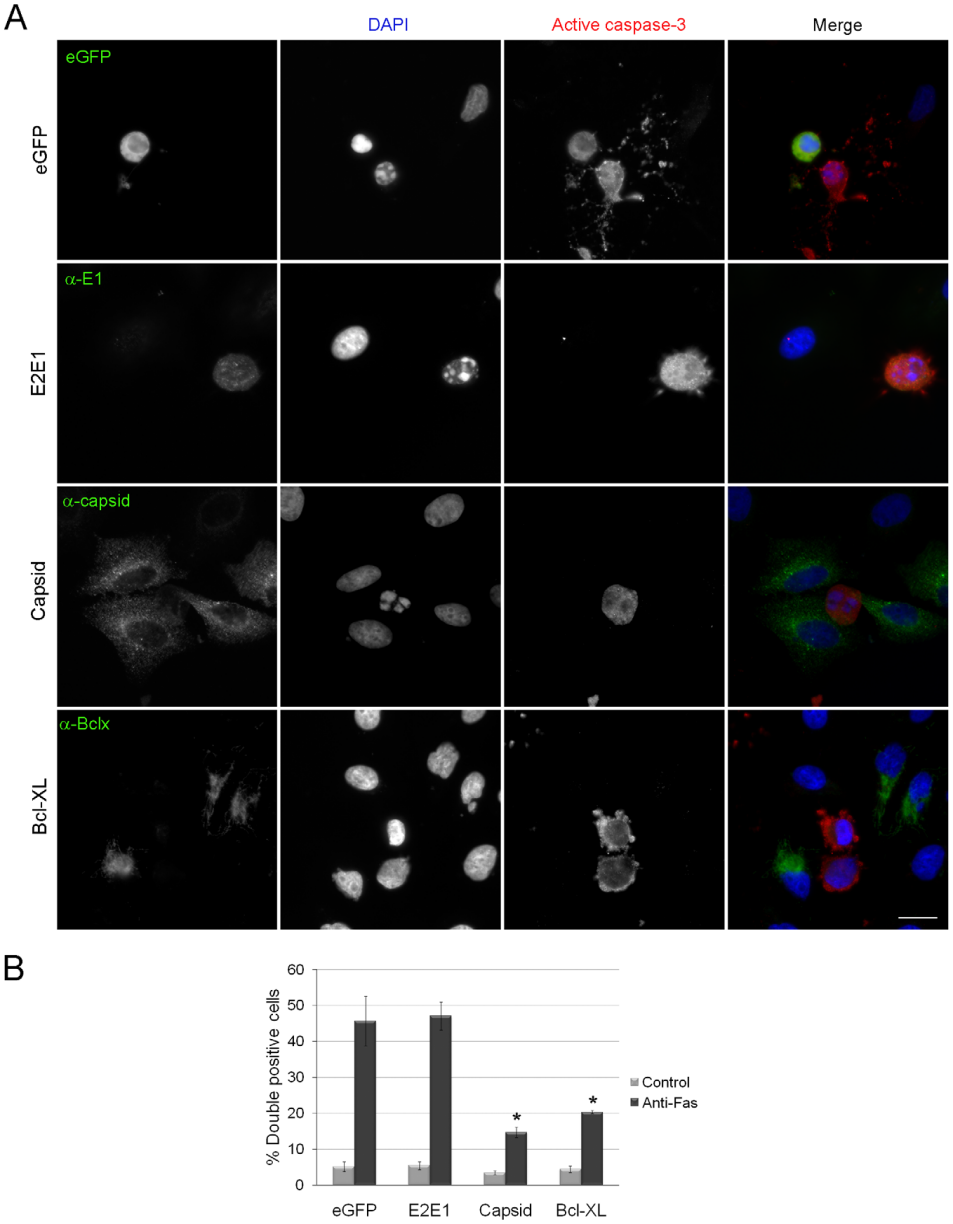
Expression of RV capsid protein blocks activation of caspase 3. A. A549 cells were transiently transfected with plasmids encoding eGFP, RV glycoproteins E2 and E1, capsid protein or the anti-apoptotic protein Bcl-XL. At forty hours post-transfection, cells were treated with anti-Fas for 6 hours after which time they were processed for immunofluorescence using rabbit anti-caspase 3 and mouse antibodies to E1, capsid or Bcl-XL. Primary antibodies were detected with donkey anti-rabbit Alexa488 and chicken anti-mouse Alexa594. For samples expressing eGFP, the rabbit anti-caspase 3 was detected with donkey anti-rabbit conjugated to Texas Red. Nuclei were counter stained with DAPI. Scale bar  = 10 µm. B. The percentages of transfected cells expressing active caspase 3 (double positive) were determined and plotted. Error bars indicate standard deviations calculated from three independent experiments in which at least 100 cells for each experiment were scored. One-way ANOVA was used to determine statistical significance. *p* = ≤0.001.

Next, we sought to determine which viral protein(s) was primarily responsible for protecting infected cells against apoptosis. Previous studies have indicated that expression of the nonstructural proteins p150 and p90 are cytotoxic [19], [20] and therefore, we focused our attention on the virus structural proteins. Plasmids encoding glycoproteins E2 and E1 or capsid, were transiently tranfected into A549 cells and at 40 hours post-transfection, cells were induced to undergo apoptosis by treatment with anti-Fas. Samples were processed for indirect immunofluorescence (Figure 2A) and the numbers of active caspase 3-positive transfectants were determined. Data in Figure 2B show that the levels of apoptosis were similar in cells expressing the viral glycoproteins E2 and E1 and the negative control protein eGFP. In contrast, expression of the RV capsid protein was just as protective against anti-Fas as the well-characterized anti-apoptotic protein Bcl-XL [21]. Compared to eGFP or E2E1 transfectants that were treated with anti-Fas, the percentage of apoptotic cells among capsid transfectants was three fold lower (Figure 2B).

We also examined whether RV infection and/or capsid expression protects Vero cells from apoptosis. This cell line is used extensively to study RV replication and similar to what was observed with A549, infection of Vero cells with RV, or transient expression of capsid protein conferred protection from staurosporine-induced apoptosis (Figure S1A, B, arrowheads). Because Vero cells do not respond to anti-Fas treatment, it was not possible to determine the effect of capsid expression on death receptor pathways. These data appear to be at odds with a previous study which reported that the RV capsid was pro-apoptotic in RK-13 cells [22]; a cell line that is exquisitely sensitive to RV-induced apoptosis [23]. Accordingly, we assayed intrinsic and extrinsic apoptotic pathways by staurosporine and anti-Fas treatment of RK-13 cells at 48 and 72 hours post-transfection. In both cases, expression of the capsid protein conferred resistance to apoptosis similar to Bcl-XL (Figure S2). Together, these data indicate that the RV capsid is an anti-apoptotic protein that protects cells from multiple apoptotic stimuli.

### Capsid protein blocks activation of the mitochondrial apoptotic pathway

**Figure 3:**
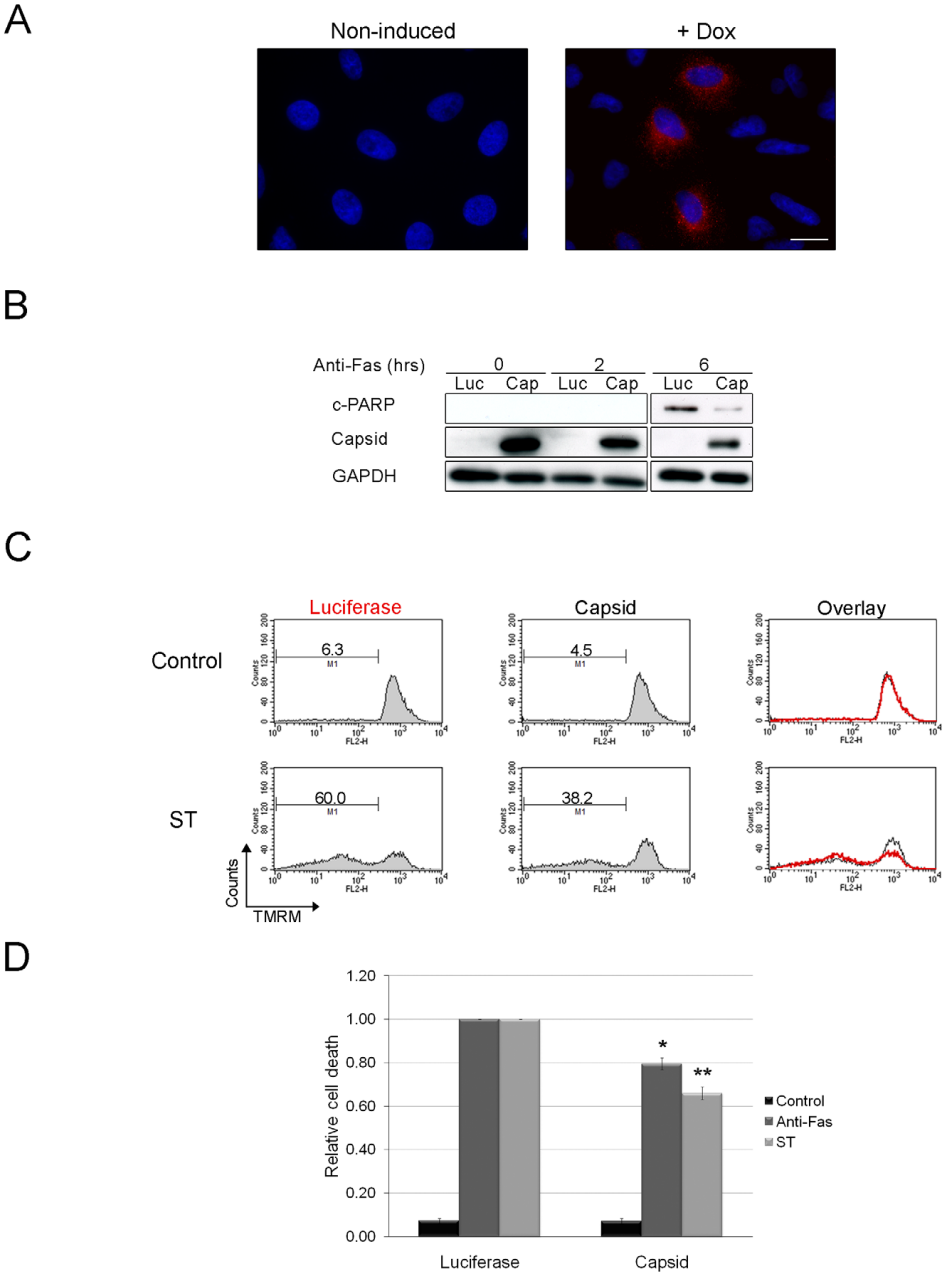
Expression of capsid in stably transduced A549 cells protects against staurosporine- and Fas-induced depolarization of mitochondria and PARP cleavage. A. A549 cells were stably transduced with a lentivirus encoding RV capsid. Following induction with doxycycline (Dox), approximately <40% of cells were found to express capsid protein as detected by indirect immunofluorescence with a mouse monoclonal antibody and chicken anti-mouse Alexa594. Nuclei were counter stained with DAPI. Scale bar  = 10 µm. B. Cells expressing capsid (Cap) or luciferase (Luc) were treated with anti-Fas for 0, 2 and 6 after which levels of cleaved PARP (c-PARP) were determined by immunoblotting. Monitoring GAPDH levels was done to illustrate comparable loading. C. Capsid protein or luciferase expression was induced with doxycycline for 48 hours and then cells were treated with staurosporine (ST) or anti-Fas antibody for 6 hours to induce apoptosis. Samples were then stained with TMRM for 30 minutes and then subjected to flow cytometric analyses. The relative level of specific cell death in each sample was calculated and plotted (D). Error bars indicate standard deviations. Student's *t*-Test was performed to determine statistical significance *p≤0.01 **p≤0.005.

We next endeavored to identify what step in apoptotic signaling was blocked by capsid protein. For these experiments, lentiviral transduction was used to create A549 cells that stably express capsid protein under the control of a doxycycline-regulated promoter. Results from indirect immunofluorescence showed that less than 50% of the polyclonal population of transduced cells expressed RV capsid following doxycyline treatment (Figure 3A). Similar to results shown in Figure 2, induction of capsid expression protected the stably transduced A549 cells against staurosporine- and Fas-mediated activation of caspase 3 (Figure S3). To further confirm that apoptotic stimuli do not activate caspases in these cells, we measured the appearance of the downstream caspase 3 substrate, cleaved Poly(ADP-ribose) polymerase (PARP). Figure 3B shows that expression of capsid protein results in decreased anti-Fas-induced cleavage of PARP compared to luciferase-expressing cells. These data indicate that capsid protects A549 cells from staurosporine and anti-Fas treatment by blocking caspase activation.

We next determined where upstream of caspase 3 activation, that capsid protein acted. Both staurosporine- and anti-Fas- can trigger apoptosis through the mitochondrial pathway, so we tested the ability of capsid protein to block depolarization of mitochondrial membranes in response to apoptotic stimuli. Doxycycline-treated A549 cells expressing capsid protein or luciferase were challenged with staurosporine or anti-Fas and then stained with the membrane potential sensitive dye TMRM. Samples were analyzed by FACS, after which the relative specific cell death levels for each sample were calculated (Figure 3C). Data in Figure 3D show that compared to luciferase, expression of capsid protein reduced the relative specific death induced through intrinsic (staurosporine) or death receptor-dependent pathways (Fas) by 20–35%. However, because less than 50% of the lentivirus-transduced cells express detectable levels of capsid protein, these numbers likely underestimate the true level of protection afforded by stable expression of the RV capsid protein.

**Figure 4:**
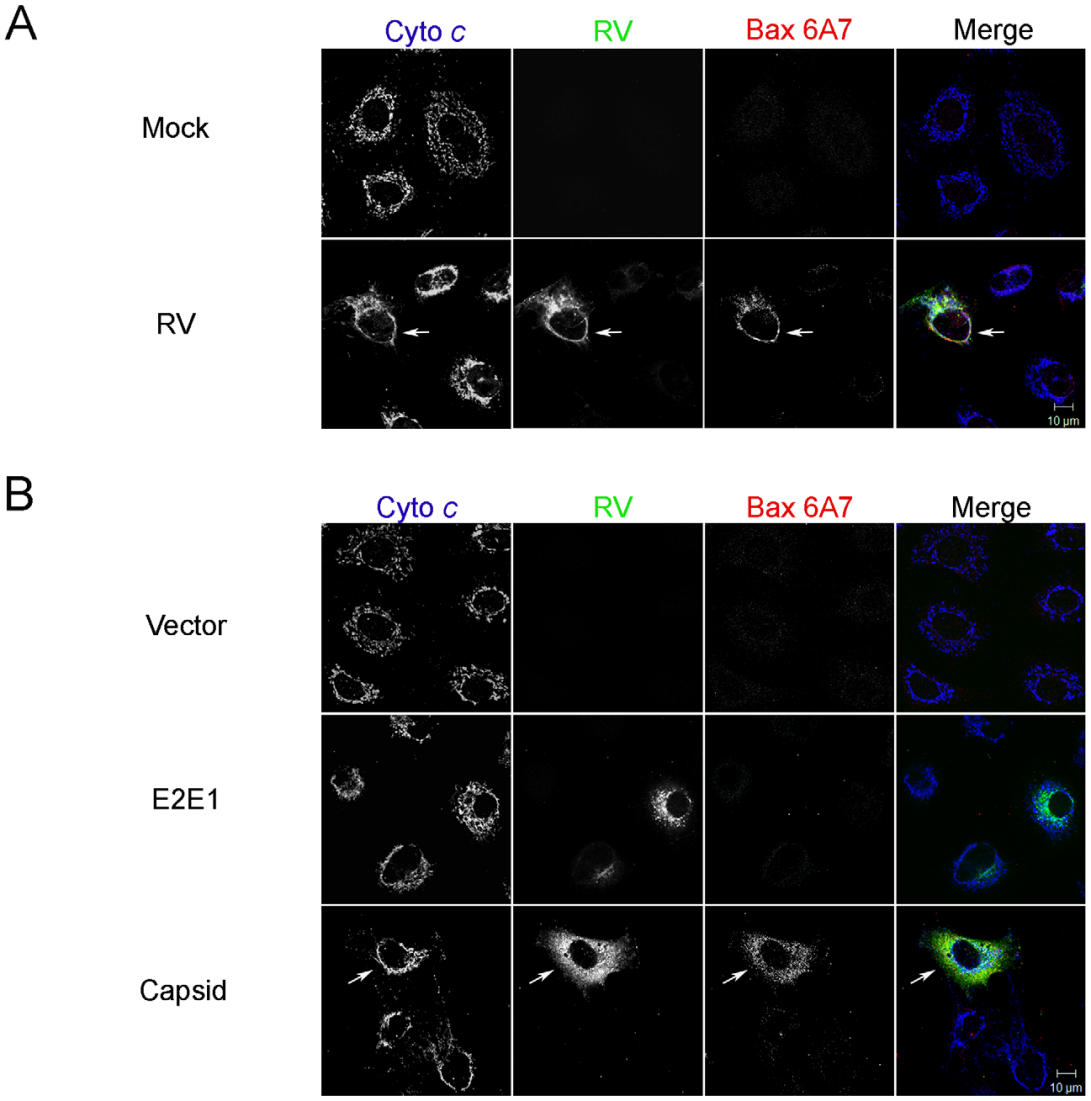
RV infection activates Bax. A. A549 cells were infected with RV (MOI = 1) and then processed for indirect immunofluorescence after 48 hours. Samples were stained with goat anti-RV, rabbit anti-cytochrome c and a mouse monoclonal (6A7) that recognizes activated Bax. Primary antibodies were detected with chicken anti-goat Alexa488, donkey anti-rabbit Alexa637, and chicken anti-mouse Alexa594. B. A549 cells were transiently transfected with plasmids encoding RV capsid, glycoproteins E2 and E1 or vector alone. After 48 hours, samples were processed for indirect immunofluorescence as described in A. Images shown are representative of three independent experiments in which at least 100 infected or transfected cells were examined. Scale bar  = 10 µm.

**Figure 5:**
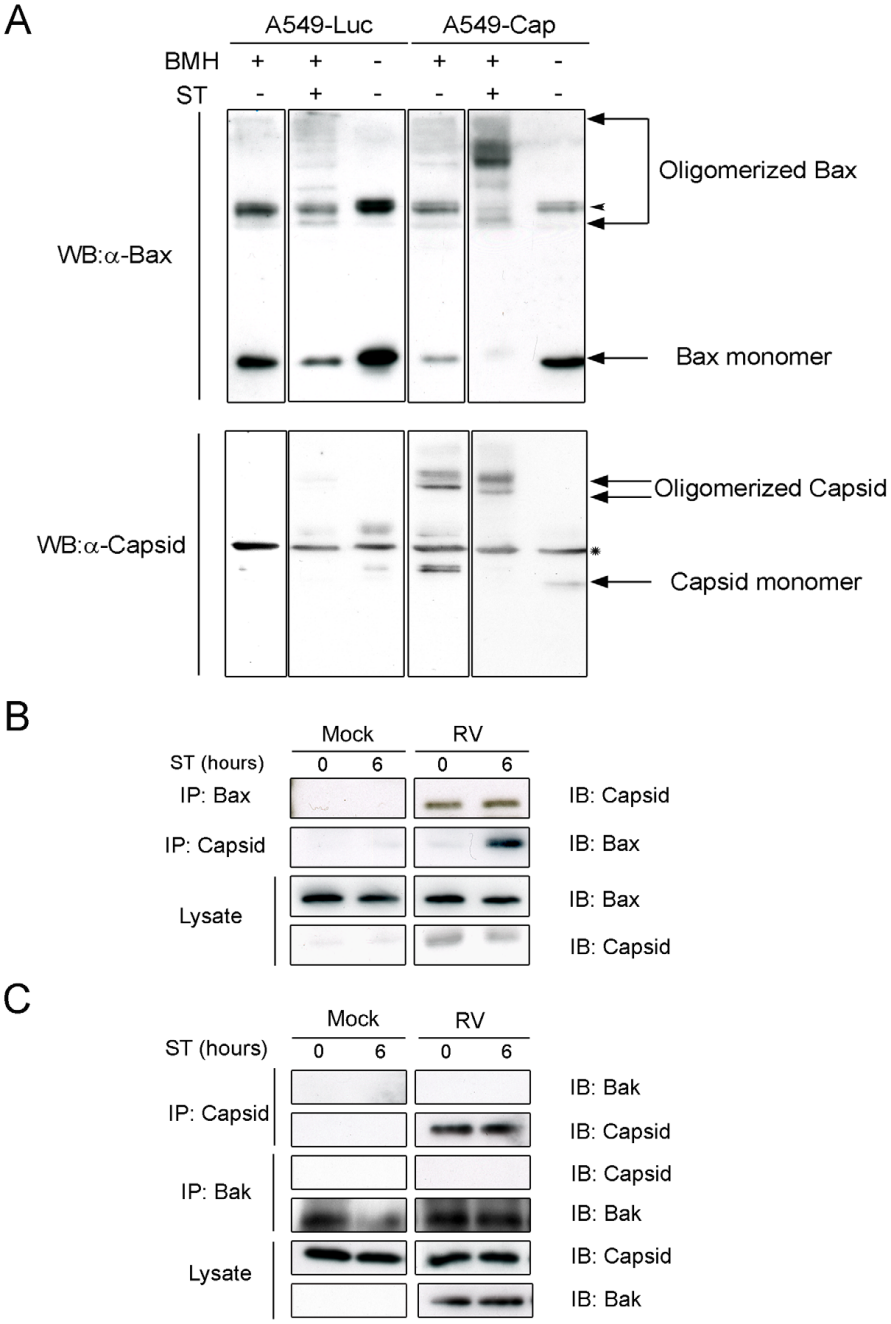
Capsid protein forms complexes with Bax. A. Stably transduced A549 cells expressing luciferase (Luc) or capsid protein (Cap) were treated with staurosporine (ST) for 6 hours. After ST treatment, crude mitochondria were isolated and then crosslinked with BMH prior to analyses by immunblotting with antibodies to capsid and Bax. The arrowhead in the upper panels denotes an SDS-resistant Bax dimer that is present in all samples. The asterisk in the lower panels denotes a non-specific protein that cross-reacts with the capsid antibody. A549 cells were infected with RV and at 42 hours, were treated with ST for 0 to 6 hours. Cell lysates were subjected to immunoprecipitation (IP) with antibodies to capsid, Bax (B) or Bak (C) followed by SDS-PAGE and immunoblotting (IB).

Bax and Bak are two key apoptotic molecules that form oligomers on mitochondria [24], [25], [26] and apoptosis occurs when the mitochondrial outer membrane is permeabilized by these pore-forming molecules [27]. Accordingly, we next focused our efforts on these Bcl-2 family members starting with Bax. Normally, Bax is an inactive monomer found in the cytosol or loosely bound to the mitochondrial outer membrane of healthy cells [28], [29]. In response to apoptotic stimuli, Bax activation is characterized by a multi-step process whereby it undergoes a conformational change [30], [31], integrates into the mitochondrial membrane [28], [32] where it forms higher order oligomers [33]. It is the large Bax oligomers that are linked to the formation of membrane pores that facilitate release of mitochondrial cytochrome *c* and downstream caspase activation [33], [34]. Of these multiple steps, Bax conformational change can be detected by immunoreactivity with a conformation-specific antibody, 6A7 [35], [36]. We observed that RV infection induces Bax conformational change, however cytochrome *c* remained associated with mitochondria (Figure 4A, arrows). Moreover, Bax conformational change as detected by 6A7 staining was evident in the majority (76%) of cells expressing capsid protein (Figure 4B arrows). In contrast, among cells expressing the viral glycoproteins E2 and E1, only 6% contained activated Bax. Despite initial stimulation of Bax, similar to infected cells, no loss of cytochrome *c* from mitochondria was observed in capsid-expressing cells. Because capsid protein stimulates Bax in a manner that does not produce functional pores that mediate efflux of cytochrome *c*, we initially thought that capsid protein blocks oligomerization of Bax. However, data in Figure 5A indicate that this is not the case. Rather, our results suggest that capsid protein and Bax form mixed large hetero-oligomers even in the absence of apoptotic stimuli. Indeed, reciprocal co-immunoprecipitation experiments confirmed that capsid forms a stable complex with Bax (Figure 5B). Staurosporine treatment enhanced the formation of the capsid:Bax hetero-oligomers but evidently did not facilitate the assembly of functional Bax pores as the cells were not apoptotic. Interestingly, we found no evidence that capsid protein binds to Bak (Figure 5C) suggesting the interaction of this viral protein with Bcl-2 family proteins is highly specific. Together, these data suggest that capsid protein and Bax form mixed oligomers that do not function as pores.

### Capsid protein protects against Bax- but not Bak-induced apoptosis

**Figure 6:**
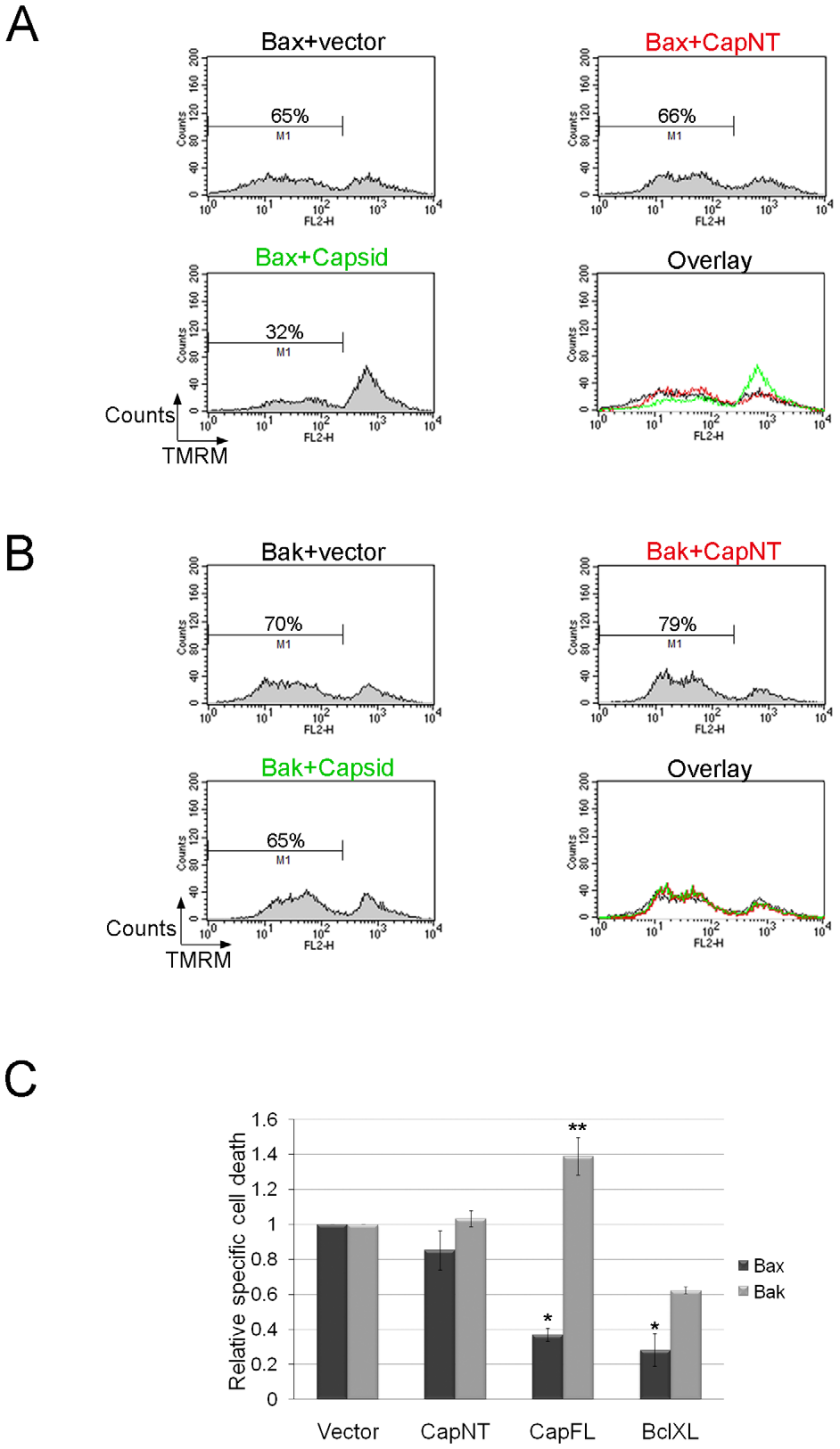
Capsid expression protects against Bax- but not Bak-induced apoptosis. A549 cells were co-transfected with plasmids encoding GFP-Bax or GFP-Bak together with plasmids encoding capsid, CapNT, Bcl-XL (not shown) or vector alone. After 24 hours, samples were stained with TMRM for 30 minutes and then subjected to flow cytometric analyses. Sample FACS plots for GFP-Bax (A) and GFP-Bak (B) transfectants are shown. C. The levels of relative specific cell death in GFP positive cells were calculated and plotted. Error bars indicate standard deviations calculated from three independent experiments. *p≤0.001, **p≤0.01.

Since capsid protein forms a complex with Bax, we next tested whether its expression could inhibit Bax-mediated apoptosis. Over-expression of either Bax or Bak induces cell death in the absence of other apoptotic stimuli [37]. A549 cells were co-transfected with plasmids encoding GFP-Bax and capsid, Bcl-XL (positive control) or vector alone (negative control) and at 24 hour post-transfection, samples were stained with the membrane-potential specific dye TMRM and then subjected to flow cytometric analyses (Figure 6A). As a second control, we transfected cells with a plasmid encoding a capsid deletion construct (CapNT) that is not targeted to mitochondria (see below). Loss of TMRM staining as a result of depolarization of mitochondrial membranes was used as the measure of apoptotic cell death. Quantitation of the data (Figure 6C) revealed that expression of capsid protein reduced the level of Bax-induced cell death by more than 60% compared to CapNT or vector alone. Similar results were observed for cells expressing Bcl-XL, a protein which has previously been shown to block the effects of Bax over-expression [38]. Data in Figure S4 show that capsid expression also protects primary human embryonic fibroblast (HEL-18) cells [17] from Bax-mediated apoptosis. The anti-apoptotic activity of capsid protein was specific to Bax as evidenced the fact that it did not attenuate Bak-mediated apoptosis (Figure 6B, C).

**Figure 7:**
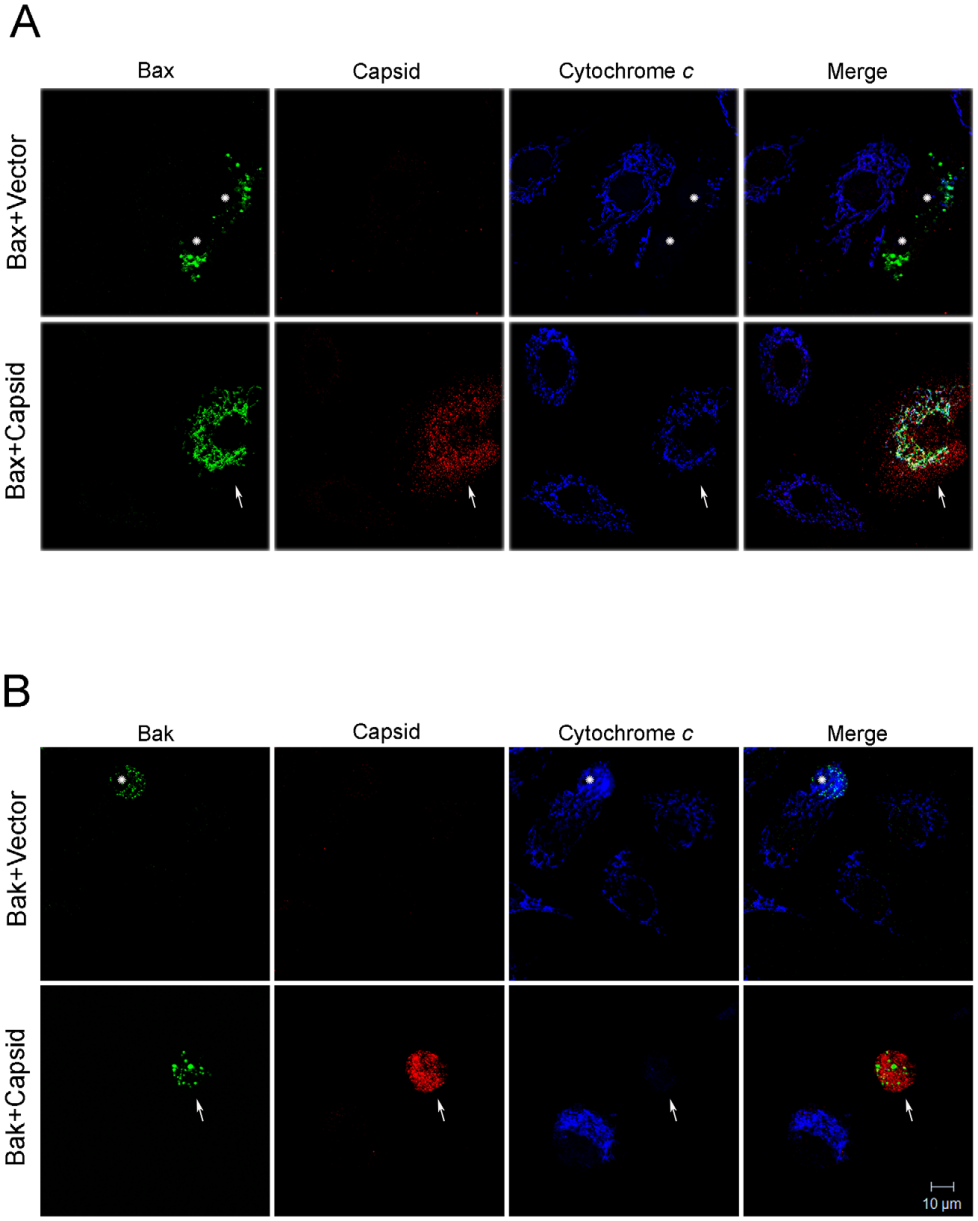
Capsid expression prevents Bax-induced release of cytochrome *c* from mitochondria. A549 cells were co-transfected with plasmids encoding capsid or vector alone together with GFP-Bax (A) or GFP-Bak (B). After 24 hours, cells were stained with mouse anti-capsid and rabbit anti-cytochrome *c*. Primary antibodies were detected with chicken anti-mouse Alexa594 and donkey anti-rabbit Alexa637. Asterisks mark Bax or Bak expressing cells that have released their mitochondrial stores of cytochrome *c* into the cytoplasm. In part A, the arrows denote a cell expressing both capsid and Bax. In this cell, it can be seen that cytochrome *c* remains associated with mitochondria. In part B, the arrowheads denote a cell expressing both capsid and Bak. However, in this case, the cell has released its mitochondrial stores of cytochrome *c* into the cytoplasm. Images shown are representative of three independent experiments in which at least 100 cells were examined. Scale bar  = 10 µm.

To further understand how capsid functions to block apoptosis, we determined whether expression of this viral protein inhibits Bax-induced release of cytochrome *c*. A549 cells were co-transfected with plasmids encoding GFP-Bax and capsid or empty vector. Localization of cytochrome *c* was monitored by fluorescence microscopy at 24 hours post-transfection. As expected, in cells expressing GFP-Bax and vector alone, there was marked loss of cytochrome *c* from mitochondria (Figure 7A, asterisks). In contrast, in cells that expressed both capsid protein and GFP-Bax, cytochrome *c* remained associated with this organelle (Figure 7A, arrows). However, consistent with data shown in Figures 5 and 6, capsid did not block GFP-Bak-induced loss of cytochrome *c* from mitochondria (Figure 7B arrows).

### Capsid:Bax interactions form the basis for the anti-apoptotic activity of capsid

**Figure 8:**
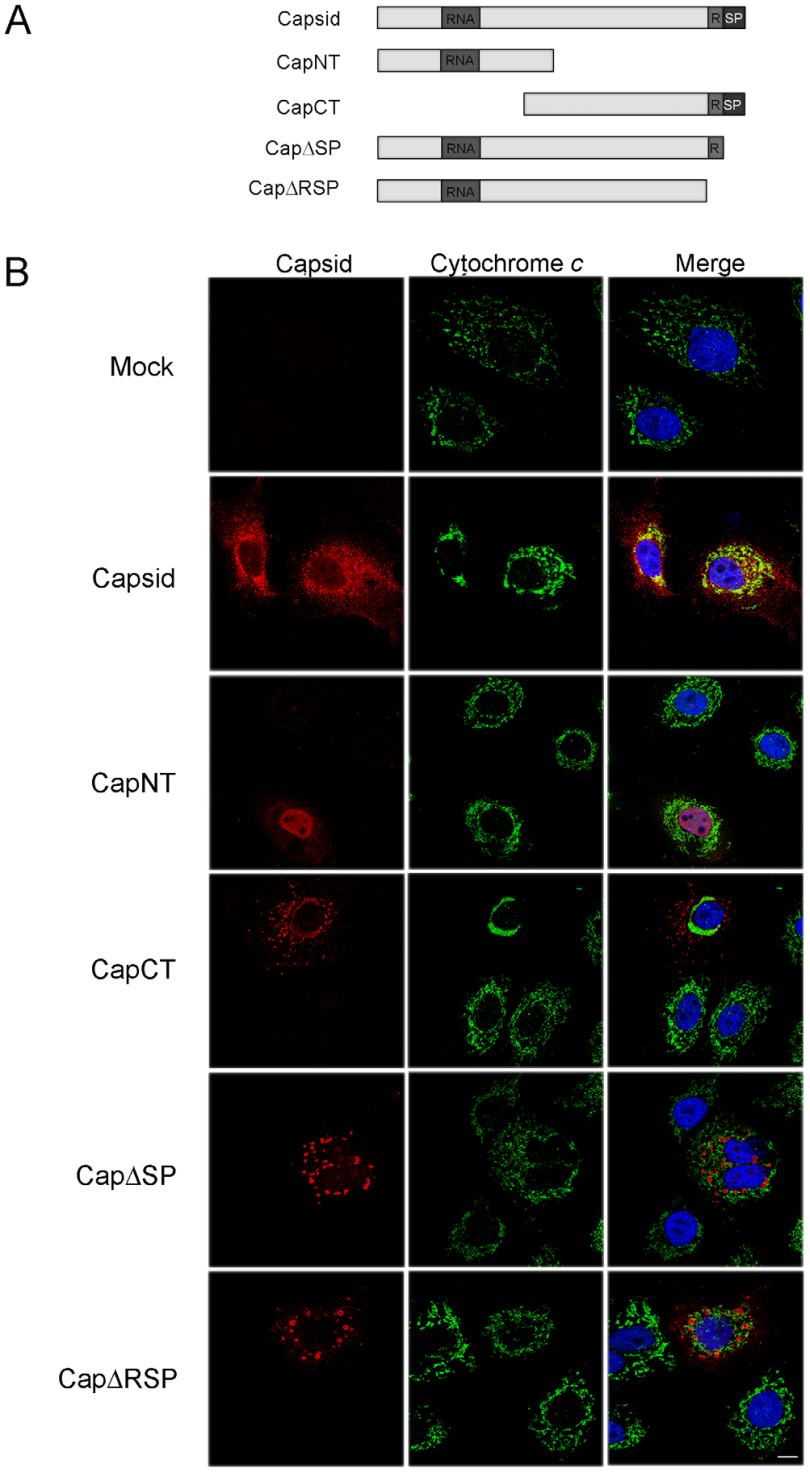
The E2 signal peptide is required for targeting of capsid protein to mitochondria. A. Schematic of capsid constructs used for transfection experiments. RNA = RNA-binding domain; R = Arginine-rich motif; SP = E2 signal peptide. Numbers denote the amino acid residues of capsid protein. B. A549 cells were transfected with plasmids encoding capsid constructs and were then processed for indirect immunofluorescence after 40 hours. With the exception of transfectants expressing CapNT, all samples were stained with mouse anti-capsid and rabbit anti-cytochrome *c* to label mitochondria. Primary antibodies were detected with chicken anti-mouse Alexa594 and donkey anti-rabbit Alexa488. CapNT was detected using goat anti-RV serum and chicken anti-goat Alexa594. Nuclei were stained with DAPI. Scale bar  = 10 µm.

Based on the assumption that association of capsid protein with mitochondria is critical for its anti-apoptotic function, we next mapped the region of capsid protein that is required for targeting to this organelle. Analyses of the RV capsid protein sequence with web-based algorithms such as PSORT II Prediction (http://psort.nibb.ac.jp/form2.html) indicated that conventional mitochondrial targeting signals are absent. We therefore constructed a series of capsid deletion mutants whose localizations were determined by expression in A549 cells (Figure 8A). From the indirect immunofluorescence data shown in Figure 8B, it can be seen that the 23 amino acid residue E2 signal peptide which forms the hydrophobic carboxyl-terminus of capsid protein, is required for association with mitochondria. Moreover, the observation that a pool of CapCT overlaps with cytochrome *c* indicates that the carboxyl-terminal region of capsid protein contains information that is sufficient for targeting to mitochondria. Intriguingly, expression of the CapCT construct caused extreme compaction of the mitochondrial network to the perinuclear region, much more so than in cells expressing full-length capsid protein.

**Figure 9:**
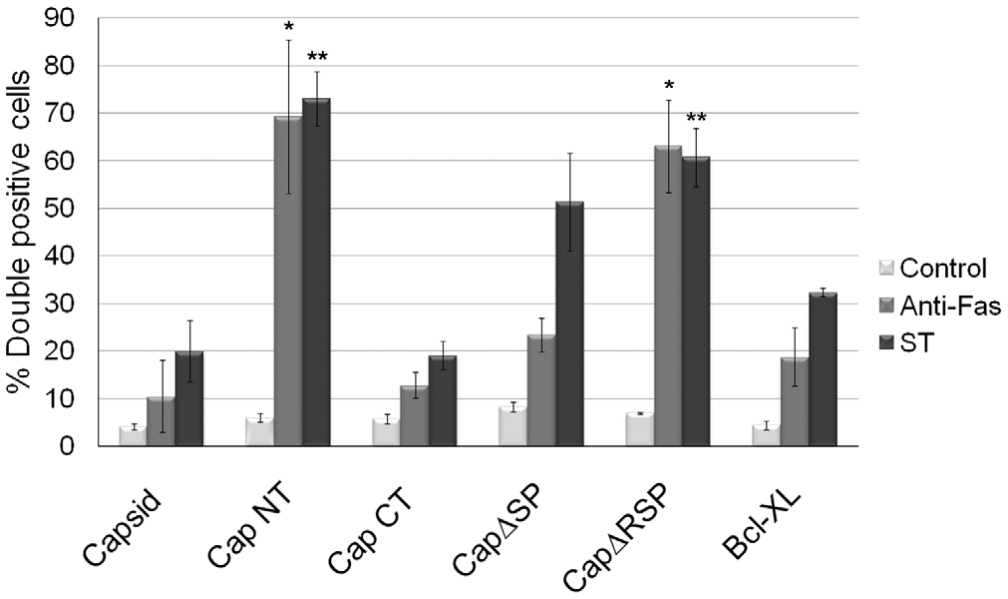
The anti-apoptotic function of capsid protein maps to the carboxyl-terminal region. A549 cells were transiently transfected with various capsid constructs or Bcl-XL and at 42 hours post-transfection, cells were treated with staurosporine or anti-Fas for 6 hours after which the numbers of transfectants that were positive for active caspase 3 (double positive) were determined by indirect immunofluorescence. Error bars indicate standard deviations calculated from three independent experiments. *p≤0.0001, **p≤0.001.

We next determined whether association of capsid with mitochondria correlated with its ability to block apoptosis. Transfected cells expressing the various capsid constructs were challenged with staurosporine or anti-Fas, and then apoptosis induction was assessed using the activated caspase 3 assay. The amino-terminal capsid construct (CapNT) neither associates with mitochondria nor protects against apoptosis (Figures 8, 9). Conversely, CapCT, a pool of which is targeted to mitochondria, protects as well as full-length capsid protein against staurosporine and anti-Fas challenge. CapΔRSP, which lacks the hydrophobic E2 signal peptide and a membrane proximal arginine-rich (R) motif, is not targeted to mitochondria and does not block staurosporine or anti-Fas-mediated induced activation of caspase 3. Interestingly, although CapΔSP does not localize to mitochondria, it did confer resistance to both Fas- and staurosporine-induced apoptosis (Figure 9). This observation suggests that the membrane-proximal R motif is important for the anti-apoptotic function of capsid. Table 1 summarizes the localization and anti-apoptotic properties of the capsid deletion mutants.

**Table 1:** Properties of Capsid mutants.

Construct	Mitochondria localization	Bax binding	Protection against anti-Fas	Protection against ST
Capsid	Yes	Yes	Yes	Yes
CapNT	No	No	No	No
CapCT	Yes	No	Yes	Yes
CapΔSP	No	Yes	Yes	Yes
CapΔRSP	No	No	No	No
CapCR5A	Yes	Yes	aND	aND

a ND =  Not determined.

**Figure 10:**
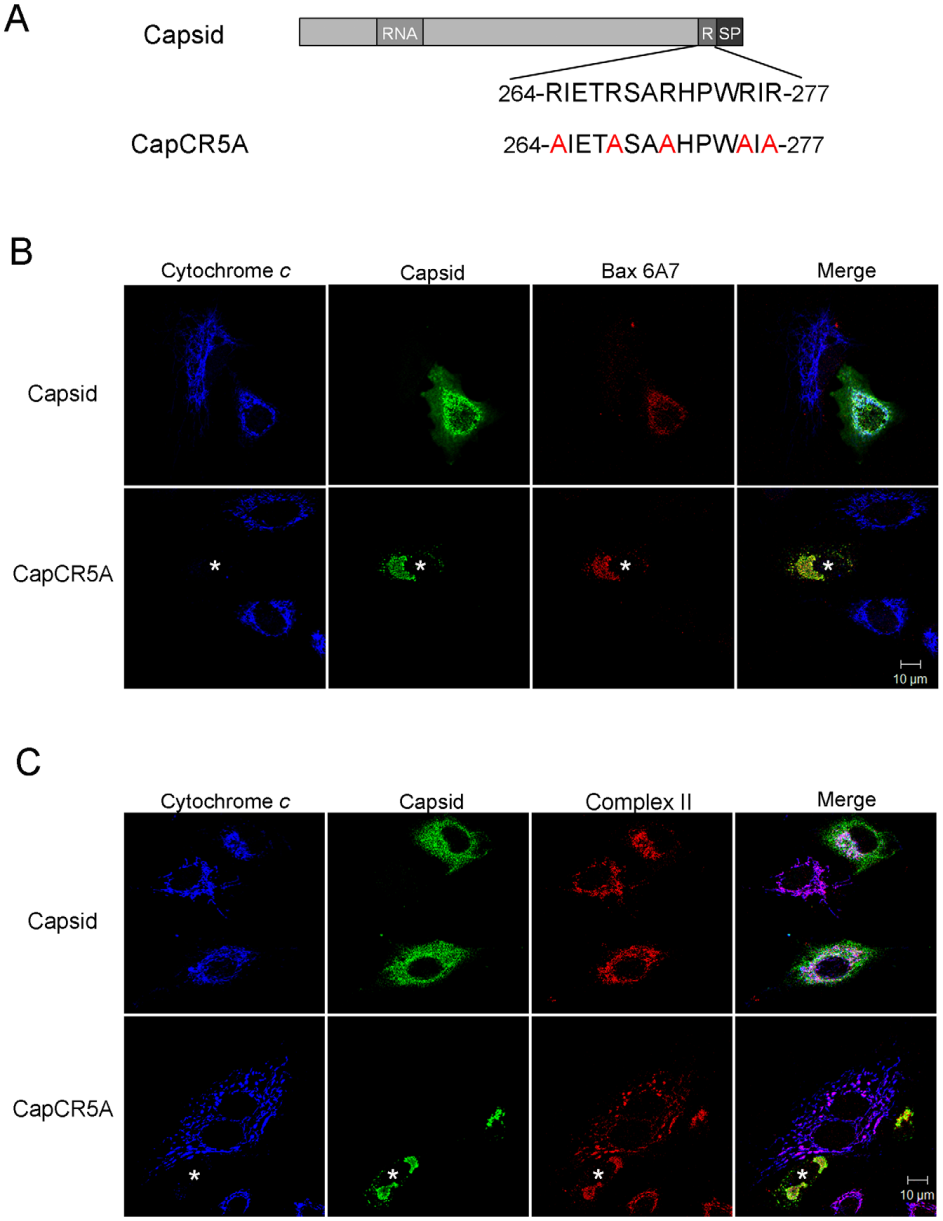
The arginine-rich motif in capsid protein is not required for targeting to mitochondria or activation of Bax. A. In the CapC5RA mutant, 5 arginine residues in the R motif were changed to alanines. RNA  =  RNA binding site; R =  arginine-rich motif; SP  =  E2 signal peptide. B. A549 cells were transfected with plasmids encoding wild type capsid or CapC5RA. At 40 hours post-transfection, cells were processed for indirect immunofluorescence using goat anti-RV, rabbit anti-cytochrome *c* and a mouse monoclonal antibody specific for activated Bax (6A7). Asterisk denotes a cell expressing CapC5RA that has loss its mitochondrial stores of cytochrome *c*. C. Samples were processed as in panel B except that a mouse monoclonal antibody to Complex II was included (instead of anti-Bax) to show that CapCR5A was targeted to mitochondria. Scale bars  = 10 µm.

**Figure 11:**
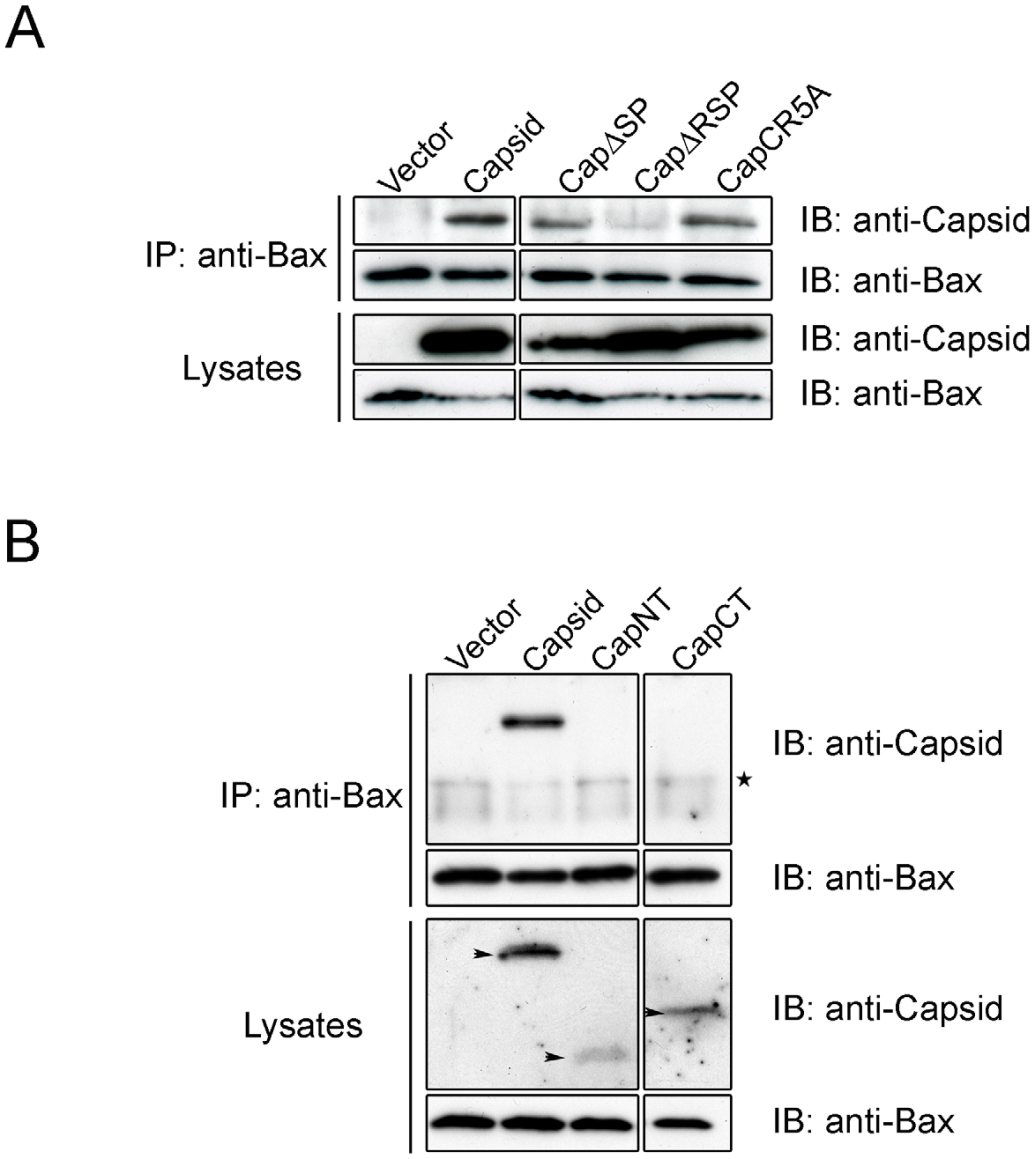
The carboxyl-terminal R motif in capsid protein is required for binding to Bax. A549 cells transiently expressing capsid, CapNT, CapCT, CapΔSP, CapΔRSP, CapC5RA or vector alone were subjected to immunoprecipitation (IP) with rabbit anti-Bax under non-denaturing conditions. Samples were resolved by 10% (A) or 15% (B) SDS-PAGE and analyzed by immunoblotting (IB) with goat anti-RV to detect capsid proteins or mouse anti-Bax. Arrowheads in the lower panel denote the various capsid constructs. The asterisk indicates IgG light chain.

To investigate if the arginine residues in the membrane-proximal R motif were important for the anti-apoptitic function of capsid protein, we created a point mutant (CapCR5A) in which five arginines in this motif were changed to alanine residues (Figure 10A). This capsid mutant was targeted to mitochondria where it activated Bax and stimulated cytochrome *c* release in the absence of apoptotic stimuli (Figure 10B and C asterisks); indicating that the arginine residues within the R domain are critical for the anti-apoptotic activity of capsid protein. Moreover, it would appear that mutation of these arginine residues unmasks an intrinsic pro-apoptotic activity of capsid protein, which may explain why it alone can stimulate Bax conformational change and membrane insertion. Next, we compared the Bax-binding ability of the CR5A mutant relative to wild type capsid and capsid deletion constructs. The observation that more CapΔSP is recovered in anti-Bax coimmunoprecipitations than CapΔRSP (Figure 11A) suggests that the R domain is important for interaction with Bax. However, ablation of the arginine residues in the R domain did not affect binding to Bax indicating that the arginine residues *per se* in this motif are not essential for interaction with Bax (Figure 11A). Binding between Bax and CapCT or CapNT was not detected in our assays (Figure 11B). Indirect immunofluorescence analyses revealed that unlike wild type capsid and CapCR5A, neither CapNT, CapCT, CapΔSP nor CapΔRSP induced the 6A7-specific conformation change in Bax (data not shown). Together, these results suggest that capsid protein employs a multi-step mechanism to block apoptosis. Specifically, binding to Bax through the R domain and/or the carboxyl terminus stimulates a conformational change in Bax; but pore formation and/or functionality is blocked by the arginines in the R motif of capsid protein.

### Recombinant viruses with mutations in the R domain are less resistant to apoptosis and are replication defective

**Figure 12:**
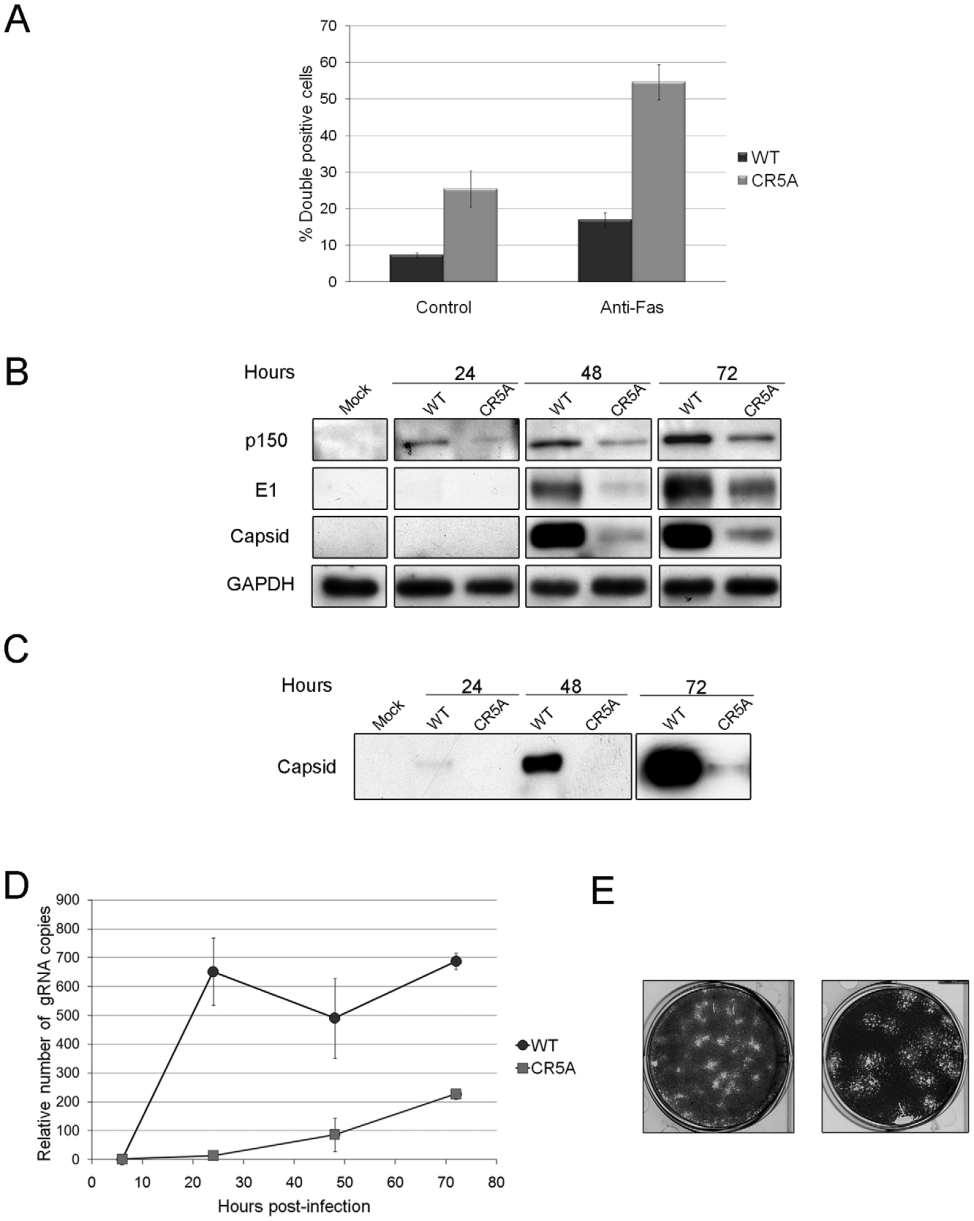
The carboxyl-terminal R motif in capsid protein is required to block apoptosis during RV infection. A. A549 cells were infected with wild type (WT) or CR5A strains of RV (MOI = 1) and 42 hours later, cells were treated with anti-Fas to induce apoptosis. Cells were processed for indirect immunofluorescence using rabbit anti-caspase 3 and mouse anti-capsid. The average number of double-positive cells for each experimental condition was determined from three independent experiments and the results were plotted. *p≤0.005, **p≤0.001. B. Vero cells were infected with WT or CR5A strains of RV (MOI = 1). At the indicated times, cell lysates (60 µg) were subjected to immunoblot analyses using antibodies to p150, capsid, E1 and GAPDH (loading control). C. Cell culture supernatants from infected Vero cells were centrifuged at 100,000× g and the pellets were subjected to immunoblot analyses using antibodies to capsid. D. Relative levels of genomic viral RNA (gRNA) in the infected Vero cells were determined by qRT-PCR at the indicated time periods. E. RK13 cells were infected with wild type WT or CR5A strains of RV and plaque assays were performed. The CR5A-derived plaques are much larger and have a spotty appearance.

We introduced the CR5A mutations into the capsid gene of a RV infectious clone in order to determine if the membrane-proximal arginine-rich (R) domain in capsid protein is required for blocking apoptosis during infection. Our hypothesis was that early onset apoptosis would result in decreased replication and virus particle production. A549 cells were infected with wild type or CR5A strains of RV and virus replication and apoptosis induction were analyzed. Data in Figure 12A show that cells infected with CR5A virus were significantly more susceptible to Fas-dependent apoptosis. Moreover, in non-treated (control) samples, the level of virus-induced apoptosis was four fold higher in cells infected with the CR5A mutant. Similar results were obtained with infected Vero cells (data not shown). Next, we compared the levels of RV proteins in CR5A and wild type (WT) RV infected cells as a function of time. Figure 12B shows that in cells that were infected with WT RV, the level of virus nonstructural (p150) and structural proteins (capsid) peaked at 72 hours. In contrast to p150 levels which were only moderately lower, steady state levels of capsid protein were dramatically lower in CR5A infected cells at all time points. To control for the possibility that CR5A capsid was unstable in the infected cells, we also determined the relative levels of another structural protein, E1. Similar to capsid protein levels in CR5A infected cells, levels of E1 were much lower than in WT virus infected cells; suggesting a defect in synthesis of structural proteins in CR5A infected cells. Consistent with this theory, data in Figure 12C show that secretion of CR5A virions is severly impaired. This was not because the CR5A capsid is misfolded as data in Figure S5 show that this mutant capsid protein functions as well as wild type capsid in driving assembly and secretion of Rubella virus-like particles.

Nonstructural proteins are translated directly from the 40S genomic RNA whereas capsid and other structural proteins are made from a subgenomic RNA. Accordingly, it is possible that virus transcription and replication are impaired in the CR5A mutant. Quantitative RT-PCR with p90 specific primers was used to determine the relative levels of genomic RNA in the WT and CR5A infected samples (Figure 12D). From these data, it can be seen that replication of viral RNA was severely affected in CR5A infected cells. This was not due to decreased infection efficiency because at six hours post-infection, there was on average >50% more genomic RNA in CR5A infected cells (Table 2). Moreover, as demonstrated by plaque assays, cells infected with CR5A virus did release infectious virus (Figure 12E). Interestingly, the CR5A plaques were larger and had a spotty appearance compared to wild type virus-produced plaques which were smaller and clearer.

**Table 2:** Relative genomic RNA levels in infected Vero cells.

	*Hours post-infection*			
*RV strain*	6	24	48	72
WT	1	623.2±116.8	336.5±138.3	818.8±28.5
CR5A	1.7±0.1	14.7±4.5	94.6±57.5	245.4±18.6

**Figure 13:**
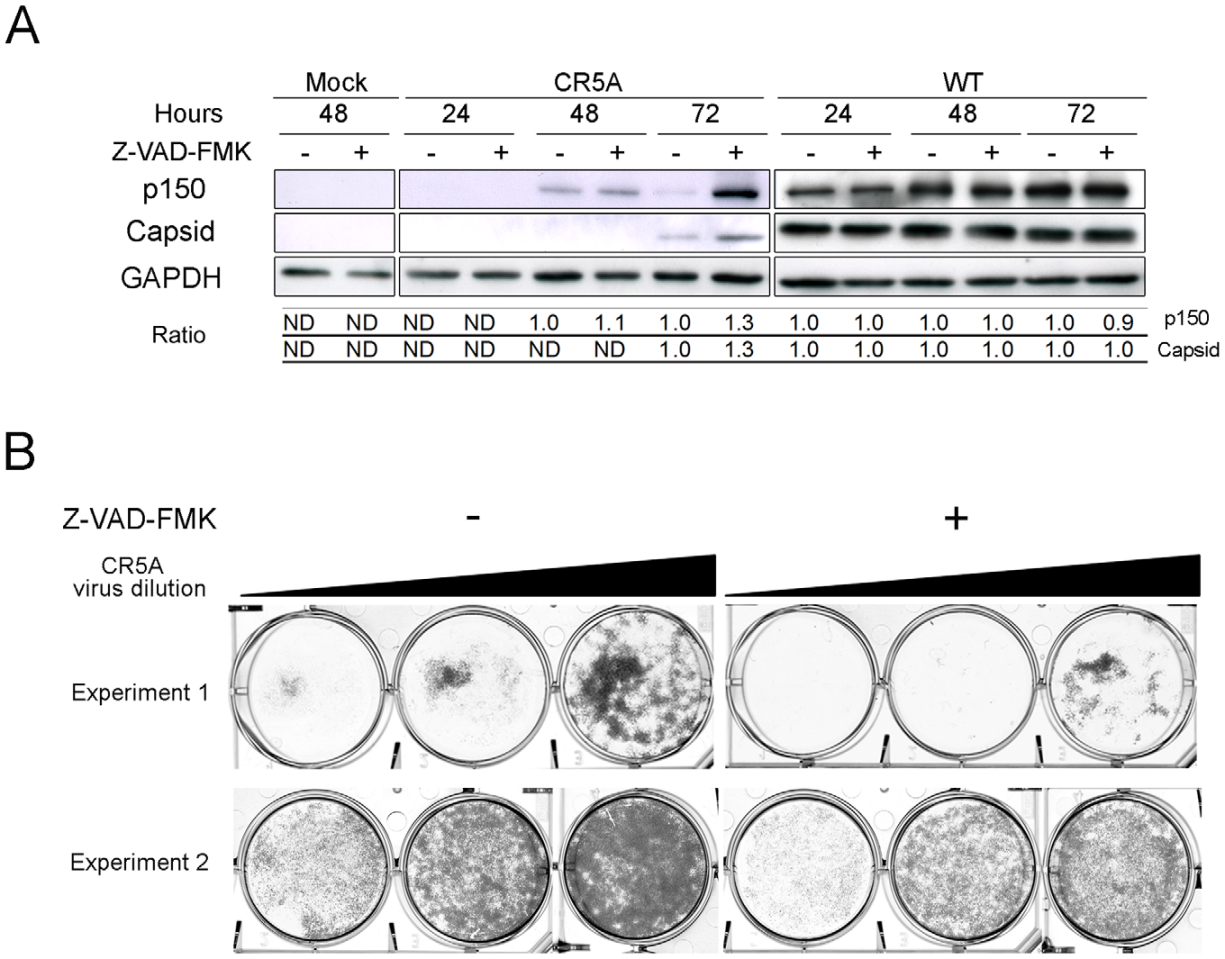
Vero cells that were infected with the CR5A mutant or wild type RV were treated with DMSO (-) or Z-VAD-FMK (50 µM) every day for up to 3 days. A. Cell lysates and culture supernatants were harvested at indicated time periods. Equal amounts of cell lysates (60 µg) were subjected to immunoblot analyses with antibodies to viral (p150 and capsid) and cellular proteins (GAPDH). At each time point, the relative signals of the p150/GAPDH and capsid/GAPDH in DMSO-treated cells were normalized to 1.0. Relative ratios of Z-VAD-FMK to DMSO samples were then determined. In some cases (ND), it was not possible to determine the relative ratios due to low signal intensity. B. Media from infected cells were serially diluted 10-fold before addition to RK-13 monolayers for plaque assays. Based on the clearing of cell monolayers, it can be seen that supernatants from CR5A infected Z-VAD-FMK-treated Vero cells contain more infectious virus.

Although data in Figure S5 indicate that Cap5RA is not misfolded, without additional investigation, we could not completely rule out the possibility that the replication defects associated with the CR5A strain virus were due to other inherent defects of the mutant capsid protein. Therefore, we attempted to artificially block apoptosis by over-expression of Bcl-XL or adding the caspase inhibitor Z-VAD-FMK to CR5A infected cells. Over-expression of Bcl-XL did not rescue the CR5A replication but this result was non-informative as further investigation revealed that this anti-apoptotic protein was unable to protect mitochondria from the effects of CapC5RA in transfected cells (data not shown). In contrast, addition of Z-VAD-FMK did have a modest effect on production of viral proteins in CR5A infected cells (Figure 13A). The effect was most pronounced at 72 hrs post-infection where levels of p150 and capsid were considerably higher in Z-VAD-FMK treated cells. In contrast, blocking caspase activity in cells that were infected with wild type RV did not appreciably alter the steady state levels of viral proteins. Finally, it can be seen from the data in Figure 13B that Z-VAD-FMK treatment had a modest effect on production of CR5A virus. Compared to CR5A-infected Vero cells treated with DMSO alone, addition of Z-VAD-FMK resulted in a modest increase in viral titers as evidenced by increased clearing of RK-13 monolayers. Together, these data are consistent with our hypothesis that the anti-apoptotic function of capsid is important for virus replication.

## Discussion

Apoptosis is a common defense mechanism used by host cells to limit the spread of viral infections and consequently, a number of viruses have developed mechanisms to disrupt programmed cell death pathways. With few exceptions, all known viral apoptosis inhibitors are accessory proteins that are encoded by DNA viruses and therefore, a great deal of effort has focused on these proteins (reviewed in [39]). Ironically, even though RNA viruses cause the vast majority of viral diseases in humans, comparatively little is known about if or how these types of viruses interfere with apoptotic signaling. Among the exceptions are picornaviruses, a number of which encode “security” proteins (leader protein and 2BC) that can block apoptosis [40], [41]. The mechanisms by which these proteins block apoptosis are not known and interestingly, caspase activation can still occur normally. These proteins may not actually prevent cell death *per se*, but rather, shift the balance toward necrotic cell death as opposed to apoptosis.

Hepatitis C virus (HCV) is known to modulate apoptotic signaling but unlike picornaviruses, this virus is not cytolytic and readily establishes persistent infections *in vivo*. HCV encodes a number of proteins that reportedly exhibit anti-apoptotic activity. For example, the nonstructural proteins NS2 and NS5A interfere with programmed cell death by different mechanisms [42], [43]. The functions of HCV structural proteins in apoptotic signaling events are less clear; in particular, the core/capsid protein. The majority of data suggest that this protein acts to induce apoptosis although a number of published studies suggest otherwise (reviewed in [44]). Similarly, with one exception [45], expression of HCV E2 glycoprotein reportedly acts in a pro-apoptotic manner [46], [47], [48]. By and large, these studies involved plasmid-based expression of individual HCV proteins and indeed the data provide much to ponder with respect how this virus interfaces with apoptotic pathways. However, it is still not clear how individual HCV proteins or those of any other RNA virus affect cell death during infection.

Multiple laboratories have reported that RV infection induces programmed cell death in a variety of cultured cell lines [17], [19], [23], [49], [50] but it is worth noting that in virtually all cases, maximum synthesis of viral macromolecules and release of virions occur well before extensive apoptosis is observed. For example, in Vero cells, robust expression of structural proteins is first detected at 16 hours post-infection and secretion of infectious virions peaks 32 hours later [51]. Conversely, late apoptotic events such as DNA fragmentation and expression of pro-apoptotic proteins p53 and p21 does not peak until 5–7 days post-infection [17]. This indicates that that the majority of programmed cell death occurs long after the peak of virus production. Consistent with these observations, we show that RV infected cells are in fact, resistant to apoptosis for at least 48 hours post-infection.

Here, we provide evidence that in addition to functioning in virus assembly, the RV capsid protein is a potent inhibitor of apoptosis. With the possible exception of HCV capsid and E2, structural proteins of RNA viruses have been found to cause apoptosis rather than prevent cell death (reviewed in [39], [44], [52]). As far as we are aware, this is the first example of a structural protein from an RNA virus that functions to block cell death pathways through interactions with Bax. Mapping studies suggest that expression of the virus nonstructural proteins is the cause of RV-induced cell death [19], [20]. Accordingly, counteracting apoptotic pathways that become activated by expression of these early proteins may be essential for efficient replication; a theory that is supported by data from reverse genetic experiments with the CR5A mutant.

Our data appear to be in disagreement with previously published data showing that capsid protein expression induces apoptosis [22]. An important distinction between the previous study and the present work is that we assayed the ability of capsid protein to protect against various apoptotic stimuli rather than assaying whether or not capsid expression induces apoptosis in the absence of stimuli. In addition, we found that capsid protein blocks apoptosis in multiple cell lines (including a primary human cell line) whereas in the afore-mentioned study, capsid protein was reported to be pro-apoptotic in RK-13 but not other cell lines. Data in the present study are also consistent with the fact that stable cell lines that express high levels of RV structural proteins (including capsid protein) are readily established in a variety of cells types [19], [53], [54]. Importantly, the results of the reverse genetic experiments suggest that the anti-apoptotic function of the capsid protein is critical for RV replication. Although we cannot absolutely rule out the possibility that mutations in the R domain of capsid directly affect its functions in replication and assembly this seems unlikely. First, the CapCR5A mutant was able to drive particle assembly and CR5A virions efficiently delivered viral RNA to host cells. Second, the region of capsid that complements p150 function in virus replication is in the amino-terminal one third of the protein [55]. Accordingly, the most logical conclusion is that the anti-apoptotic role of capsid protein is necessary to promote survival of the host cell during the long replication cycle. To our knowledge, this has never been demonstrated before for an RNA virus but it is tempting to speculate that other slowly replicating RNA viruses employ similar mechanisms to avoid killing infected cells.

**Figure 14:**
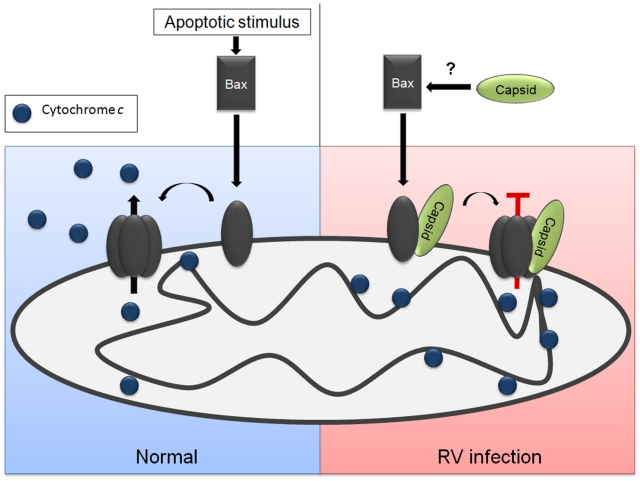
Model for how capsid blocks Bax-dependent apoptosis. Apoptotic stimuli can induce a conformational change in Bax, which is followed by stable membrane association and oligomerization. The Bax oligomers serve as pores that faciliate efflux of cytochrome *c* from the mitochondria to the cytoplasm where it initiates downstream apoptotic signaling through the apoptosome. The RV capsid protein binds to Bax before or after it is translocated to mitochondria. Interaction with capsid is followed a conformational change in Bax and subsequent hetero-oligomer formation. However, the Bax-capsid oligomers do not allow efflux of cytochrome *c* from the mitochondria and apoptosis is blocked.

Although capsid protein may interfere with apoptosis by more than one mechanism, because the Bax-dependent pathway is a critical feature of mitochondrial apoptosis in most human cell types, interfering with the pore-forming ability of this protein is likely the key anti-apoptotic function of capsid protein. Binding of capsid protein to Bax induces a major conformational change, which interestingly, seems to promote activation and oligomerization of Bax. It is not clear if this phenomenon is related to the anti-apoptotic activity of capsid or if it is an inconsequential effect of complex formation with Bax. Figure 14 depicts a model in which capsid protein interferes with formation of functional Bax pores. In some critical aspects, the RV capsid protein may function analogously to the cytomegalovirus accessory protein vMIA, a putative Bcl-2 homolog that forms mixed oligomers with Bax [56]. However, confirmation of this theory is dependent upon determining the structure of the RV capsid protein.

Mapping studies localized the anti-apoptotic activity to the carboxyl-terminal region of capsid protein. The E2 signal peptide, which is required for membrane association of capsid protein [57], [58], is also essential for targeting to mitochondria but not for blocking apoptosis. Conversely, while the membrane proximal arginine-rich (R) motif in capsid is dispensable for targeting to mitochondria, it is required for protection from intrinsic and extrinsic apoptotic stimuli (Table 1). The R motif (RSARHPWRIR) of RV capsid bears remarkable similarity to the Bax-binding motif (RRHRFLWQRR) in vMIA [59] which is critical for blocking Bax- but not Bak-dependent apoptosis [60]. Despite the apparent similarities between vMIA and RV capsid protein, one difference worth noting is that the arginine-rich motif in capsid is not required for binding to Bax.

As mentioned above, it is possible that capsid protein blocks apoptosis through other mechanisms, at least one of which does not involve Bax. For example, CapCT, which neither binds to nor activates Bax, protects cells from staurosporine and Fas-dependent apoptosis. However, unlike full-length capsid, CapCT does not protect cells from Bax over-expression. Capsid binds two other pro-apoptotic proteins p32 and Par-4 [61] and through sequestration into non-functional complexes, it is possible that the functions of these proteins in apoptotic signaling are mitigated. Although we have no direct evidence to support this theory, binding to membrane-associated capsid protein may prevent Par-4 from engaging in pro-apoptotic complexes in the nucleus or cell surface [62], [63], [64]. Finally, we showed that translocation of the capsid-binding pro-apoptotic protein p32 into mitochondria is inhibited by RV infection [12]. Because targeting of p32 to mitochondria is critical for its function in programmed cell death pathways [65], [66], reducing the levels of mitochondrial p32 would be expected to reduce apoptosis.

To summarize, we describe a novel mechanism by which a viral capsid protein potently blocks apoptosis. Our data suggest that this function of capsid is important for virus replication and it is also tempting to speculate that establishing and/or maintaining persistent infections *in vivo* also requires this activity. RV is known to persistently infect lymphocytes and capsid-dependent inhibition of Fas-dependent apoptosis may allow the virus to disseminate through the body using apoptosis-resistant lymphocytes as conduits. It will be of interest to examine proteins from other slowly replicating RNA viruses to determine if capsids or other proteins double as inhibitors of apoptosis.

## Materials and Methods

### Reagents

The following reagents were purchased from the respective suppliers: Protein A and G Sepharose from GE Healthcare Bio-Sciences Corp (Princeton, NJ); General lab chemicals from Sigma Chemical Co. (St. Louis, MO); Media and fetal bovine serum (FBS) for cell culture from Life Technologies-Invitrogen, Inc. (Carlsbad, CA); A549, HEK 293T, Vero, and RK-13 cells from the American Type Culture Collection (Manassas, VA.). Hel-18 cells [17] were obtained from Dr. Eva Gonczol, (National Center for Epidemiology, Budapest, Hungary).

### Mammalian cell culture and virus infection

A549 and HEK 293T cells were cultured in Dulbecco's minimal essential medium (high glucose) containing 10% FBS, 2 mM glutamine, 1 mM HEPES, and antibiotics. Vero cells were cultured in Dulbecco's minimal essential medium (high glucose) containing 5% FBS, 2 mM glutamine, 1 mM HEPES, and antibiotics. RK-13 cells were cultured in minimum essential medium containing 10% FBS, 2 mM glutamine, 1 mM HEPES, 0.1 mM non-essential amino acids, and antibiotics. Hel-18 cells were cultured in RPMI-1640 medium containing 10% FBS, 2 mM glutamine, 1 mM HEPES, 0.1 mM non-essential amino acids, and antibiotics. Cells were incubated at 37°C in a humidified atmosphere with 5% CO_2_. RV stocks were diluted with cell culture media and then added to cells that had been washed with PBS. Cells were incubated with the virus (1 ml/35 mm dish) for 4 hours at 35°C after which time the inoculum was replaced with normal growth media. Infected cultures were kept at 35°C until experimental analyses.

To investigate the effect of blocking apoptosis on virus replication, Vero cells were infected with M33 (wild type) or CR5A strains of RV (MOI: 1) in the presence of 50 µM pan-caspase inhibitor Z-VAD-FMK (Promega, Madison, WI) which was initially made as a 20 mM stock solution in dimethyl sulfoxide (DMSO). Z-VAD-FMK or control vehicle (DMSO) was added to infected cells every 24 hrs. Samples were processed at the indicated time points and virus titers were determined by plaque assay [67].

### Plasmid construction and lentivirus production

Plasmids for expression of pCMV5-Capsid, pCMV5-CapCT, pCMV5-CapΔSP and pCMV5-E2E1 have been described previously [58], [67], [68]. An expression plasmid encoding amino acid residues 1–152 of capsid (CapsidNT) was constructed by polymerase chain reaction (PCR) using a forward primer with a *Eco*RI site and a Kozak consensus ribosome-binding site ( 5′-CGGAATTCGCCACCATGGCTTCCACTACCCCCATCACC-3′′) and a reverse primer with a *Bam*HI site and stop codon (5′-CGCGCGGATCCCTAGGCCTCAGTGGGTGC-3′). The restriction sites are underlined in the primer sequences. The CapΔRSP cDNA which encodes amino acid residues 1–267 of capsid, was constructed by PCR using the forward *Eco*RI and Kozak site-containing forward primer (5′-CGGAATTCGCCACCATGGCTTCCACTACCCCCATCACC-3′′) and a reverse primer with a *Bam*HI site and stop codon (5′-CGCGCGGATCCCTACTCGGTGGTGTGAGGG-3′). The template cDNA for the CapNT and CapΔRSP PCR reactions was pCMV5-capsid. The CapC5RA expression plasmid was prepared by PCR using a forward primer containing an *Eco*RI site and a Kozak site (5′-TCACGGAATTC-3′) and a reverse primer containing a *Bam*HI site (5′-TCAGGATCCCTAGGCGCGCGCGGTGC-3′). The template DNA was pBRM33-CR5A. The CapsidNT, CapsidΔRSP, and CapsidC5RA cDNAs were resulting products were sublconed into the *Eco*RI and *Bam*HI sites of the mammalian expression vector pCMV5 [69] to produce pCMV5-CapNT, pCMV5-CapΔRSP and pCMV5-CapCR5A respectively.

For establishing capsid and luciferase expressing stable cell lines, the Lenti-X-tet-On advanced inducible expression system (Clontech Laboratories, La Jolla, CA) was utilized. A cDNA encoding full-length capsid was amplified by PCR using a forward primer with a *Bam*HI site and Kozak consensus ribosome binding site (5′-TAGGATCCGCCACCATGGCTTCCACTACCCCCATCACC-3′) and a reverse primer with a *Eco*RI site (5′-GGCCGAATTCCTAGGCGCGCGCGGTGC-3′) respectively, where the restriction sites are underlined. The DNA used as a template was pCMV5-capsid. The PCR product was digested with *Bam*HI and *Eco*RI, and subcloned into the pLVX-Tight-Puro vector. Lentivirus-production in HEK 293T and transduction of A549 cells were performed as per the manufacturer's instructions. At 48 hours post-transduction, cells were split 1∶2 into medium containing G418 (500 µg/ml) and puromycin (0.5 µg/ml). Surviving cells were tested for inducible expression of capsid by indirect immunofluorescence and immunoblot analyses. The resulting polyclonal stable cell lines A549-Luciferase and A549-Capsid were maintained in media containing G418 (250 µg/ml) and puromycin (0.25 µg/ml). To induce capsid or luciferase gene expression doxycycline (1 µg/ml) was added to the culture medium.

### Construction of RV strain with mutations in Arginine-rich domain

Codons for arginine-to-alanine mutations in the C-terminus of capsid were introduced into the RV M33 infectious clone (pBRM33) [70] by a two step cloning procedure. A 421 base pair synthetic fragment (Epoch BioLabs Inc, Sugarland, TX) containing five arginine-alanine substitutions (R264, 268, 271, 275, 277) was used to replace the analogous region in pCMV5-24S [68]. The resulting plasmid was named pCMV5-24S-CR5A. Next, the *Bsr*GI/*Bam*HI fragment from pCMV5-24S CR5A was used to replace the analogous region (*Bsr*GI/*Bam*HI) in pBRM33 resulting in the infectious clone pBRM33-CR5A.

### Quantitative PCR

Total RNA samples were isolated with TRI Reagent (Ambion) from Vero cells infected with M33 (wild type) or CR5A strains of RV (MOI: 1) according to the manufacturer's instructions. Prior to the RT-PCR reaction, 1 µg of total RNA was treated with 2 U of amplification grade DNase I (Invitrogen) as per manufacturer's recommendations. The DNase I-treated RNA samples were reverse transcribed to single-stranded cDNA using qScript Flex cDNA synthesis kit and Oligo (dT)_20_ primer (Quanta Biosciences, Gaithersburg, MD) as per manufacture's instructions.

Quantitative PCR reactions were conducted on a MX3005P thermocycler (Stratagene, La Jolla, CA) using a PerfecCTa SYBR green supermix low Rox real-time PCR kit (Quanta Biosciences). Reactions were carried out by triplicate in a total volume of 25 µl containing 5 µl of cDNA and 0.2 µM of each oligonucleotide primer. Primers used to amplify RV nucleotides 5520–5706 from the RV p90 non-structural protein coding region of the RV genome were as follow: RV-F (5′-AGGTCATGTCTCCGCATTTC-3′) and RV-R (5′-GTCCCGAGTAGCAAGGGTCT-3′). The amplification cycles for p90 samples consisted of an initial denaturating cycle at 95°C for 3 min, followed by 40 cycles of 15 s at 95°C, 30 s at 58°C, and 20 s at 72°C. Fluorescence was quantified during the 58°C annealing step, and the product formation was confirmed by melting curve analysis (57°C to 95°C). As an internal control, levels of the house keeping gene product cyclophilin A determined. Amplification was performed using the following primers, CYP-F (5′- TCCAAAGACAGCAGAAAACTTTCG-3′) and CYP-R (5′-TCTTCTTGCTGGTCTTGCCATTCC-3′). The amplification cycles for Cyclophilin A consisted of an initial denaturating cycle at 95°C for 3 min, followed by 40 cycles of 15 s at 95°C, 20 s at 60°C, and 40 s at 72°C. Fluorescence was quantified during the 60°C annealing step, and the product formation was confirmed by melting curve analysis (57°C to 95°C).

Quantification of the samples was performed using the two standard curves method [71], and the relative amount of RV genomic RNA was normalized to the relative amount of Cyclophilin A mRNA. Three independent PCR analyses were performed for each sample.

### Immunoprecipitation and immunoblotting

A549 cells (1×10^5^) in 35 mm culture dishes were infected with the M33 strain of RV (MOI = 2) and then incubated for 48 hours at 35°C prior to lysis. Alternatively, A549 cells were transiently transfected with pCMV5-capsid, pCMV5-CapNT, pCMV5-CapCT, pCMV5-CapΔSP, pCMV5-CapΔRSP or pCMV5-CapCR5A using Lipofectamine 2000 (Invitrogen). Cells were lysed in 1% (wt/vol) CHAPS, 150 mM NaCl, 50 mM Tris, pH 8.0 containing Complete EDTA-free protease inhibitors (Roche) or 1% NP-40, 150 mM NaCl, 2 mM EDTA, 50 mM Tris, pH 7.4 containing protease inhibitors. Cell lysates were clarified by centrifugation at 10,000× g for 10 minutes at 4°C. Immunoprecipitation was performed with clarified lysates and 1 µg/ml of mouse monoclonal anti-Bax6A7 (Sigma), or 1∶1000 of rabbit polyclonal anti-capsid serum (7W7), or 2 µg/ml of rabbit anti-Bak (Millipore) antibodies overnight at 4°C with rotation. Fifteen microliters of protein A or protein G sepharose (50% suspension) were added and then samples were rotated for 1 hour at 4°C before washing; twice with lysis buffer and once with PBS. Proteins were eluted from the beads by boiling in protein gel sample buffer, separated by SDS-PAGE, and then transferred to polyvinylidene fluoride (PVDF) membranes (Immobilon-P Millipore, Bedford, MA). Membranes were incubated for 1 hour at room temperature with the following antibodies and dilutions: 1∶1000 rabbit anti-RV capsid (7W7) [61],1∶1000 mouse anti-capsid (H15C22), 1∶1000 goat anti-RV (Meridian Life Science Inc, Saco, Maine), 1∶1000 rabbit anti-Bak (Cell Signaling), 1∶1000 rabbit anti-Bax (Abcam) or 1∶5000 mouse anti-Bax (YTH-2D2, Trevigen Inc, Gaithersburg, MD). To detect E1 glycoprotein by immunoblotting, it was necessary to perform SDS-PAGE under non-reducing conditions. E1 was detected using a 1∶1000 dilution of a mouse monoclonal antibody (H2C213) obtained from Abbott Labs (Abbott Park, IL). After three washes with Tris-Buffered-Saline-Tween (TBS-T), the membranes were incubated with either goat anti-rabbit, goat anti-mouse or rabbit anti-goat horseradish peroxidase-conjugated IgG (Bio-Rad Hercules, CA) for 1 hour. Membranes were washed four times with TBS-T and immunoreactive proteins were detected using Supersignal West Pico chemiluminescent substrate (Pierce Biotechnology, Rockford, IL) followed by exposure to X-ray film (Fuji Photo Film Co, LTD, Tokyo, Japan).

### PARP cleavage assay

A549-Capsid or A549-Luciferase cells were cultured in the presence of doxycycline and after 36 hours, anti-human Fas activating clone CH11 antibody (0.12 µg/ml) (Millipore, Temecula, CA) and cycloheximide (10 µg/ml) were added to the cultures for 6 hours. Cells were then lysed in 1% NP-40 buffer containing a cocktail of protease inhibitors. The lysates were centrifugated at 10,000× *g* for 10 min at 4°C, and protein concentrations were determined by BCA protein assay (Pierce Biotechnology, Rockford, IL) using bovine serum albumin as a standard. Equivalent amounts of protein (60 µg) from each lysate were resolved in 8% SDS-PAGE and transferred to PVDF membranes followed by immunoblotting with mouse monoclonal anti-cleaved PARP (Asp214) clone 19F4 antibody (Cell Signaling).

### Indirect immunofluorescence microscopy

A549 and Vero cells cultured on glass coverslips were infected with RV (MOI = 1) or transiently transfected with pCMV5-capsid or pCMV5-CapNT or pCMV5-CapCT or pCMV5-CapΔSP or pCMV5-CapΔRSP or pCMV5-E2E1 or pcDNA3.1-Bcl-XL, and peGFP-Bax or peGFP-Bax (Gift of Dr. Michele Barry, University of Alberta) using Lipofectamine 2000 (Invitrogen). After 24 or 48 hours post-infection or post-transfection as indicated, cells were fixed in 4% paraformaldehyde for 20 min, followed by quenching with PBS containing 50 mM ammonium chloride. Cell membranes were permeabilized with PBS containing 0.2% Triton-X-100 for 5 min before incubation with primary and secondary antibodies. All the washes were done in PBS containing 0.1 mM CaCl_2_ and 1 mM MgCl_2_.

RV proteins were detected with rabbit anti-capsid (7W7), mouse anti-capsid (H15C22), mouse-anti E1 (H2C213), goat anti-RV, or human anti-RV (GB) which has been described previously. Mitochondria were detected using rabbit anti-cytochrome *c* (from Dr. L. Berthiaume, University of Alberta) or with a mouse anti-complex II monoclonal antibody (Mitosciences, Eugene, OR). Activated isoforms of Bax and capsase 3 were detected with a Bax-specific mouse monoclonal antibody 6A7 (Abcam) or caspase 3-specific rabbit monoclonal antibody (BD Pharmingen) respectively. Primary antibodies were detected with Alexa Fluor 594 chicken anti-mouse, Alexa Fluor 488 donkey anti-rabbit, Alexa Fluor 488 donkey anti-mouse, Alexa fluor 637 Donkey anti-rabbit and/or Alexa 594 goat anti-rabbit (Molecular Probes, Invitrogen, Carlsbad, CA). Coverslips were mounted onto microscope slides using ProLong Gold antifade reagent with 4'-6-Diamidino-2-phenylindole (Molecular probes, Invitrogen). Samples were then examined using Zeiss 510 confocal microscope or a Zeiss Axioskop2 microscope equipped with a CoolSNAP HQ digital camera (Photometrics).

### Isolation of mitochondria and Bax cross-linking

A549-Cap or A549-Luciferase cells were cultured for 36 hours in the presence of doxycycline, followed by incubation with 1 µM staurosporine (Sigma-Aldrich) or anti-Fas antibody (0.12 µg/ml) and cycloheximide (10 µg/ml) for 6 hours. Cells were then homogenized in ice-cold mitochondria isolation buffer containing 200 mM mannitol, 70 mM sucrose, 10 mM Hepes, and 1 mM EGTA (pH: 7.5) using a dounce homogenizer with a loose fitting pestle. Unbroken cells and nuclei were pelleted by centrifugation at 500× *g* for 10 min at 4°C. The supernatants were then centrifuged at 10,000× *g* for 20 min at 4°C to obtain crude mitochondrial pellets that were cross-linked with 10 mM bis(maleimido)hexane (BMH; Thermo Scientific) for 30 min at room temperature. Samples then were separated on 4–12% polyacrylamide gels and then processed for immunobloting with rabbit polyclonal antibodies to Bax antibody (Abcam, Cambridge, MA) and capsid (7W7).

### Measurement of mitochondrial membrane potential by flow cytometry

Expression of capsid or luciferase in A549-cap or A549-luc cells respectively was induced with doxycycline for 36 hours, followed by incubation with staurosporine (1 µM) or anti-Fas antibody (0.12 µg/ml) to induce apoptosis. Cells then were stained with 0.2 µM Tetramethylrhodamine methyl ester (TMRM) (Invitrogen, Molecular probes) for 30 min at 37°C before analyses by flow cytometry (FACScan, Becton Dickinson). For each sample, 10,000 events were acquired. Data were analyzed using CellQuest software. The percentage of killing was calculated as the number of TMRM-negative cells divided by the total number of cells, and standard deviations were determined from three independent experiments.

For Bax or Bak killing assays, A549 or Hel-18 cells were transfected with peGFP-Bax or peGFP-Bak together with pCMV5, pCMV5-CapNT, or pCMV5-capsid using Lipofectamine 2000 or Lipofectamine LTX respectively (Invitrogen). After 24 hours, cells were stained with 0.2 µM TMRM for 30 min at 37°C prior to analyses by two-color flow cytometry. TMRM fluorescence was detected through the FL-2 channel equipped with a 585-nm filter and eGFP fluorescence was measured using the FL-1 channel equipped with a 489-nm filter. Data were acquired on 10,000 eGFP-positive cells per sample, and analysis was performed using CellQuest software. The relative specific cell death was calculated as the number of eGFP-positive TMRM-negative cells divided by the total number of eGFP positive cells. Standard deviations were generated from three independent experiments.

### Rubella virus-like particle assay

Vero cells (1×10^5^/35 mm dish) were transiently transfected with pCMV5-capsid or pCMV5-CapCR5A and pCMV5-E2E1 using Lipofectamine 2000. Assembly and secretion of RV-like particles was assayed after 48 hours of transfection as described elsewhere [58].

### Statistical analyses

Data from FACS and indirect immunofluorescence-based apoptosis assays were subjected to statistical analyses (student's *t* test or one-way analysis of variance (One-way ANOVA)) using Predictive Analytics Software (version 17.0.3) (SPSS Inc, Chicago, Il).

## Supporting Information

Figure S1 Vero cells that have been infected with RV or that transiently express capsid protein are resistant to apoptosis. A. Vero cells were infected with RV (MOI  =  1) or transiently transfected with plasmids encoding capsid, eGFP, E2 and E1 or Bcl-XL (B). After 40 hours, cells were treated with staurosporine (ST) for 6 hours. Samples were then processed for indirect immunofluorescence using rabbit anti-caspase 3 and mouse anti-capsid. Primary antibodies were detected with donkey anti-rabbit Alexa488 and chicken anti-mouse Alexa594. Nuclei were counter stained with DAPI. Arrowheads indicate RV infected, capsid or Bcl-XL-expressing cells that are caspase 3 negative. Images shown are representative of at least three independent experiments in which at least 100 cells were examined. Scale bar  =  10 μm.
(1.56 MB TIF)

Figure S2 Expression of capsid protein in RK-13 cells blocks apoptosis. RK-13 cells were transiently transfected with plasmids encoding eGFP, E2 and E1, capsid or Bcl-XL. A. At 42 and 66 hours post-transfection, cells were treated with staurosporine (ST) for 6 hours after which the numbers of transfectants that were positive for active caspase 3 (double positive) were determined by indirect immunofluorescence. * Differences are statistically significant according to oneway ANOVA with 95% confidence interval. B. At 42 hours post-transfection, cells were treated with anti-Fas for 6 hours after which the numbers of transfectants that were positive for active caspase 3 (double positive) were determined by indirect immunofluorescence. A minimum of 100 transfectants were analyzed per sample. Error bars indicate standard deviations calculated from three independent experiments. *p*  =  ≤ 0.001
(0.26 MB TIF)

Figure S3 Expression of capsid protein in stably transduced A549 cells protects against staurosporine- and Fas-induced activation of capsase 3. A. A549 cells were stably transduced with lentiviruses encoding RV capsid or luciferase (control). Expression of capsid and luciferease was induced with doxycycline for 48 hours after which cells were treated with staurosporine (ST) or anti-Fas for 6 hours to induce apoptosis. Samples were then processed for indirect immunofluorescence using rabbit anti-caspase 3 and mouse anti-capsid. Primary antibodies were detected with donkey anti-rabbit Alexa488 and chicken anti-mouse Alexa594. Nuclei were counter stained with DAPI. Scale bar  =  10 μm. B. The percentages of active capsase 3-positive cells were determined from three independent experiments in which at least 100 cells for each experiment were scored. Error bars indicate standard deviations calculated from three independent experiments. *p≤ 0.01, **p≤ 0.01
(0.89 MB TIF)

Figure S4 Expression of capsid protein protects primary human cells from Bax-induced apoptosis. HEL-18 cells cells were co-transfected with plasmids encoding GFP-Bax together with plasmids encoding capsid, CapNT, or vector alone. After 24 hours, samples were stained with TMRM for 30 minutes and then subjected to flow cytometric analyses. A. Sample FACS plots for GFP-Bax transfectants are shown. B) The levels of relative specific cell death in 5,000 GFP positive cells were calculated and plotted. Error bars indicate standard deviations calculated from three independent experiments.
(0.40 MB TIF)

Figure S5 The CapCR5A mutant is fully functional for assembly of rubella virus like-particles (RLP). Vero cells were transfected with plasmids encoding vector; WT capsid alone (Capsid); or a plasmid encoding glycoproteins E2 and E1 with Capsid or CapCR5A. After 48 hours, RLPs recovered from the pre-cleared media by centrifugation at 100,000 x g were detected by immunoblotting with a rabbit polyclonal antibody.
(0.18 MB TIF)
