# Supplementary figures and images for: The Rubella Virus Capsid Is an Anti-Apoptotic Protein that Attenuates the Pore-Forming Ability of Bax
Source: PLoS Pathog. 2011 Feb 17;7(2):e1001291. doi: 10.1371/journal.ppat.1001291 (PMC3040668; doi:10.1371/journal.ppat.1001291)

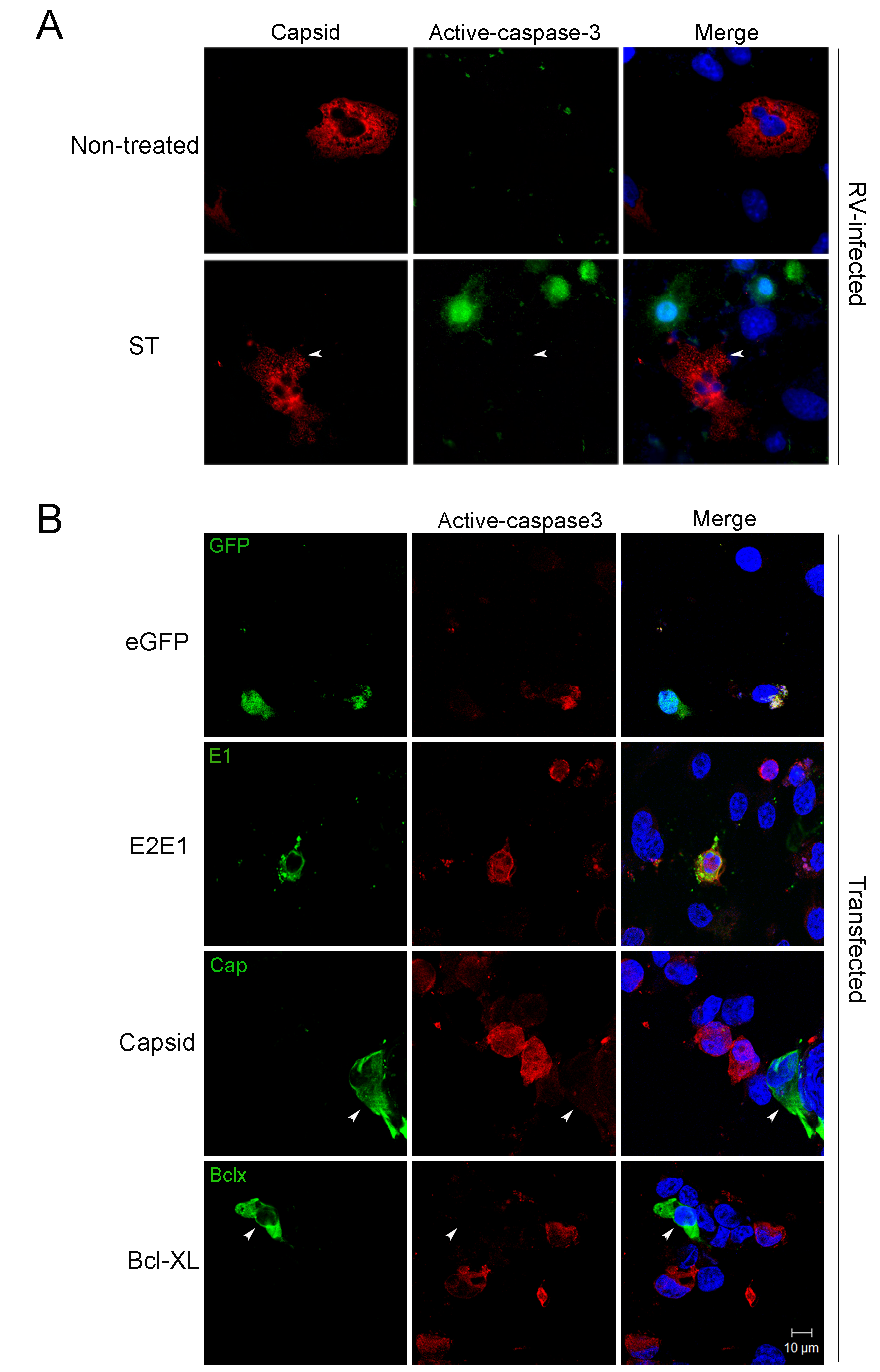

Supplement: Figure S1 — Vero cells that have been infected with RV or that transiently express capsid protein are resistant to apoptosis. A. Vero cells were infected with RV (MOI = 1) or transiently transfected with plasmids encoding capsid, eGFP, E2 and E1 or Bcl-XL (B). After 40 hours, cells were treated with staurosporine (ST) for 6 hours. Samples were then processed for indirect immunofluorescence using rabbit anti-caspase 3 and mouse anti-capsid. Primary antibodies were detected with donkey anti-rabbit Alexa488 and chicken anti-mouse Alexa594. Nuclei were counter stained with DAPI. Arrowheads indicate RV infected, capsid or Bcl-XL-expressing cells that are caspase 3 negative. Images shown are representative of at least three independent experiments in which at least 100 cells were examined. Scale bar = 10 μm. (1.56 MB TIF) [file ppat.1001291.s001.tif]

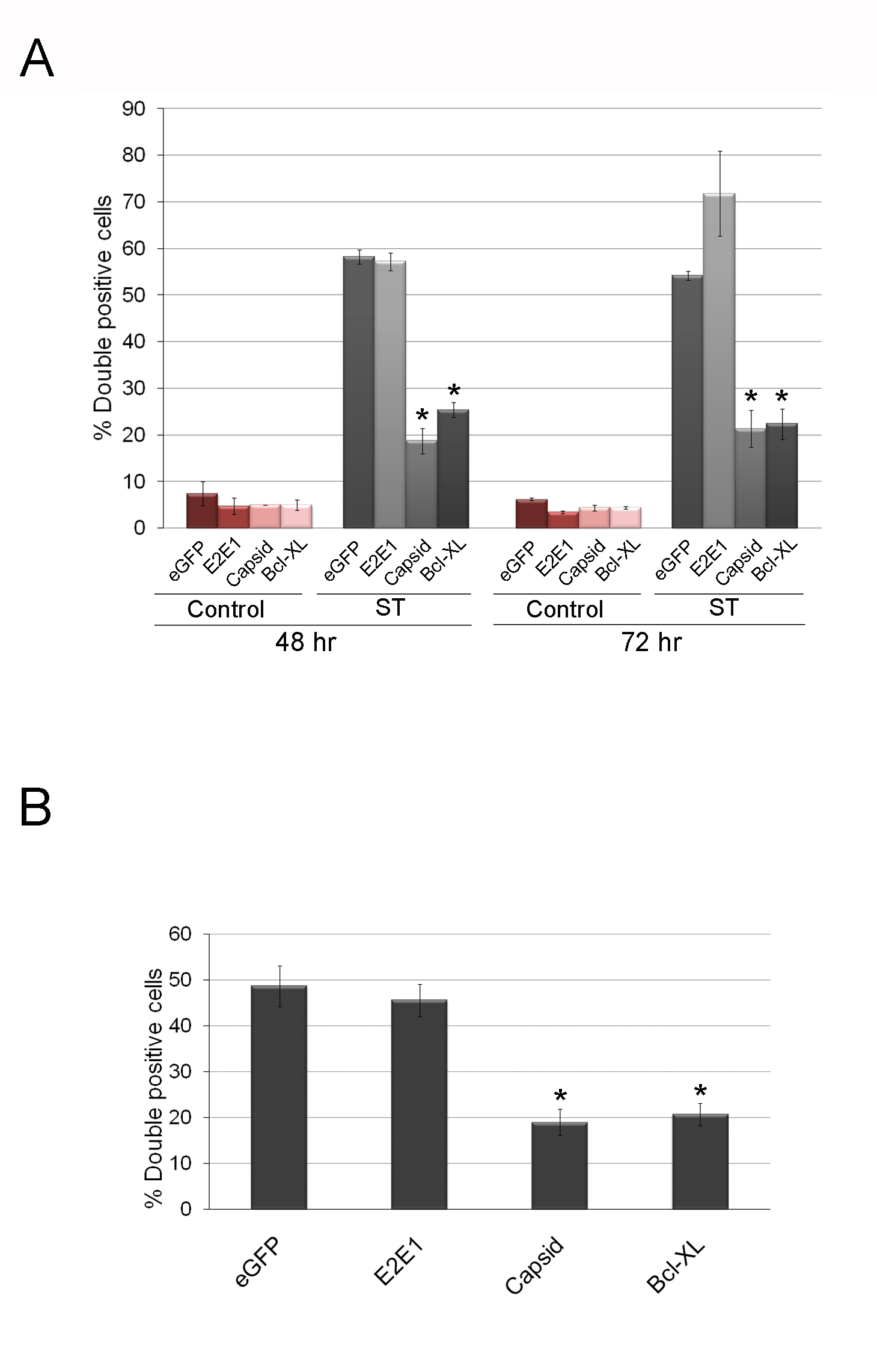

Supplement: Figure S2 — Expression of capsid protein in RK-13 cells blocks apoptosis. RK-13 cells were transiently transfected with plasmids encoding eGFP, E2 and E1, capsid or Bcl-XL. A. At 42 and 66 hours post-transfection, cells were treated with staurosporine (ST) for 6 hours after which the numbers of transfectants that were positive for active caspase 3 (double positive) were determined by indirect immunofluorescence. * Differences are statistically significant according to oneway ANOVA with 95% confidence interval. B. At 42 hours post-transfection, cells were treated with anti-Fas for 6 hours after which the numbers of transfectants that were positive for active caspase 3 (double positive) were determined by indirect immunofluorescence. A minimum of 100 transfectants were analyzed per sample. Error bars indicate standard deviations calculated from three independent experiments. p = ≤ 0.001 (0.26 MB TIF) [file ppat.1001291.s002.tif]

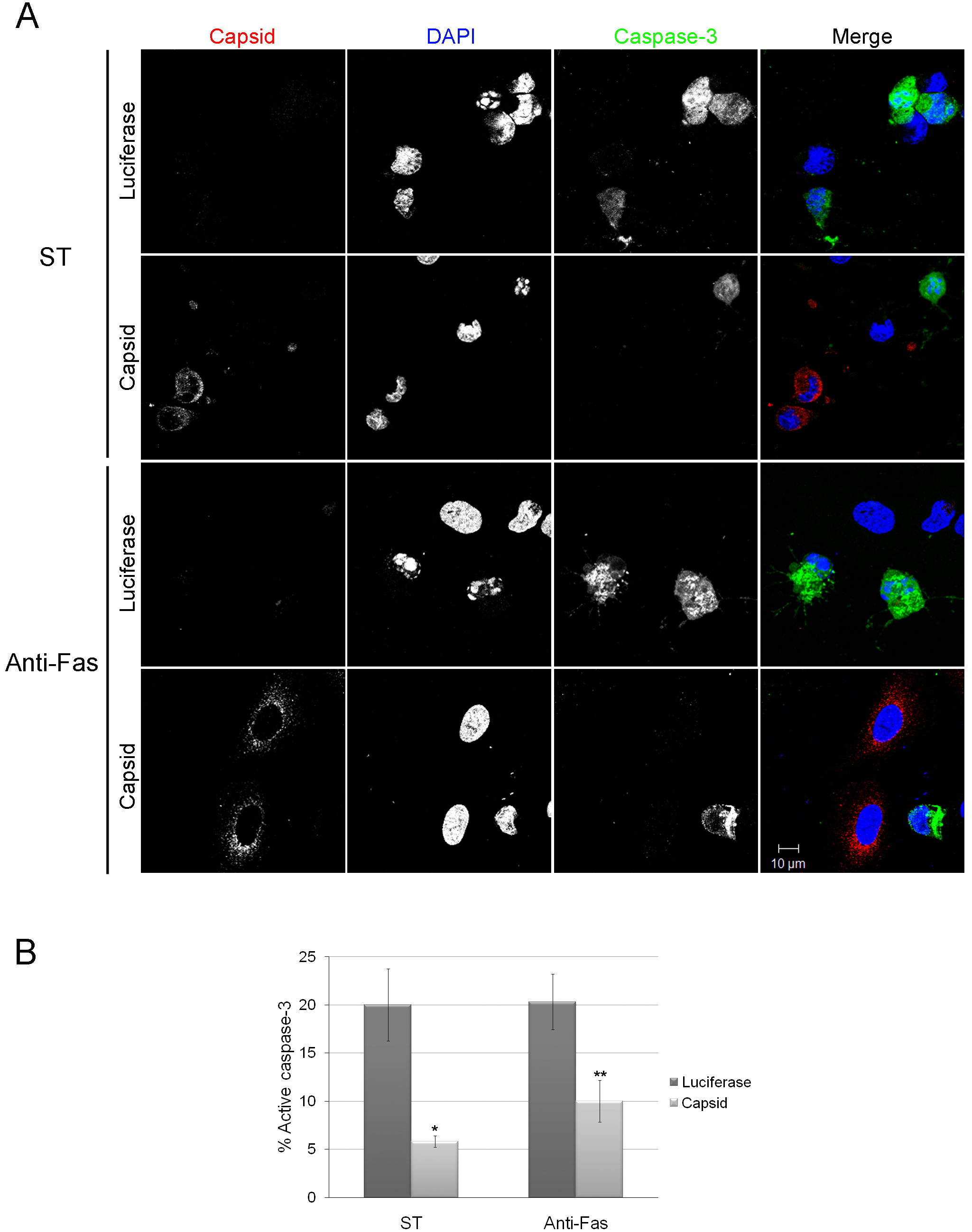

Supplement: Figure S3 — Expression of capsid protein in stably transduced A549 cells protects against staurosporine- and Fas-induced activation of capsase 3. A. A549 cells were stably transduced with lentiviruses encoding RV capsid or luciferase (control). Expression of capsid and luciferease was induced with doxycycline for 48 hours after which cells were treated with staurosporine (ST) or anti-Fas for 6 hours to induce apoptosis. Samples were then processed for indirect immunofluorescence using rabbit anti-caspase 3 and mouse anti-capsid. Primary antibodies were detected with donkey anti-rabbit Alexa488 and chicken anti-mouse Alexa594. Nuclei were counter stained with DAPI. Scale bar = 10 μm. B. The percentages of active capsase 3-positive cells were determined from three independent experiments in which at least 100 cells for each experiment were scored. Error bars indicate standard deviations calculated from three independent experiments. *p≤ 0.01, **p≤ 0.01 (0.89 MB TIF) [file ppat.1001291.s003.tif]

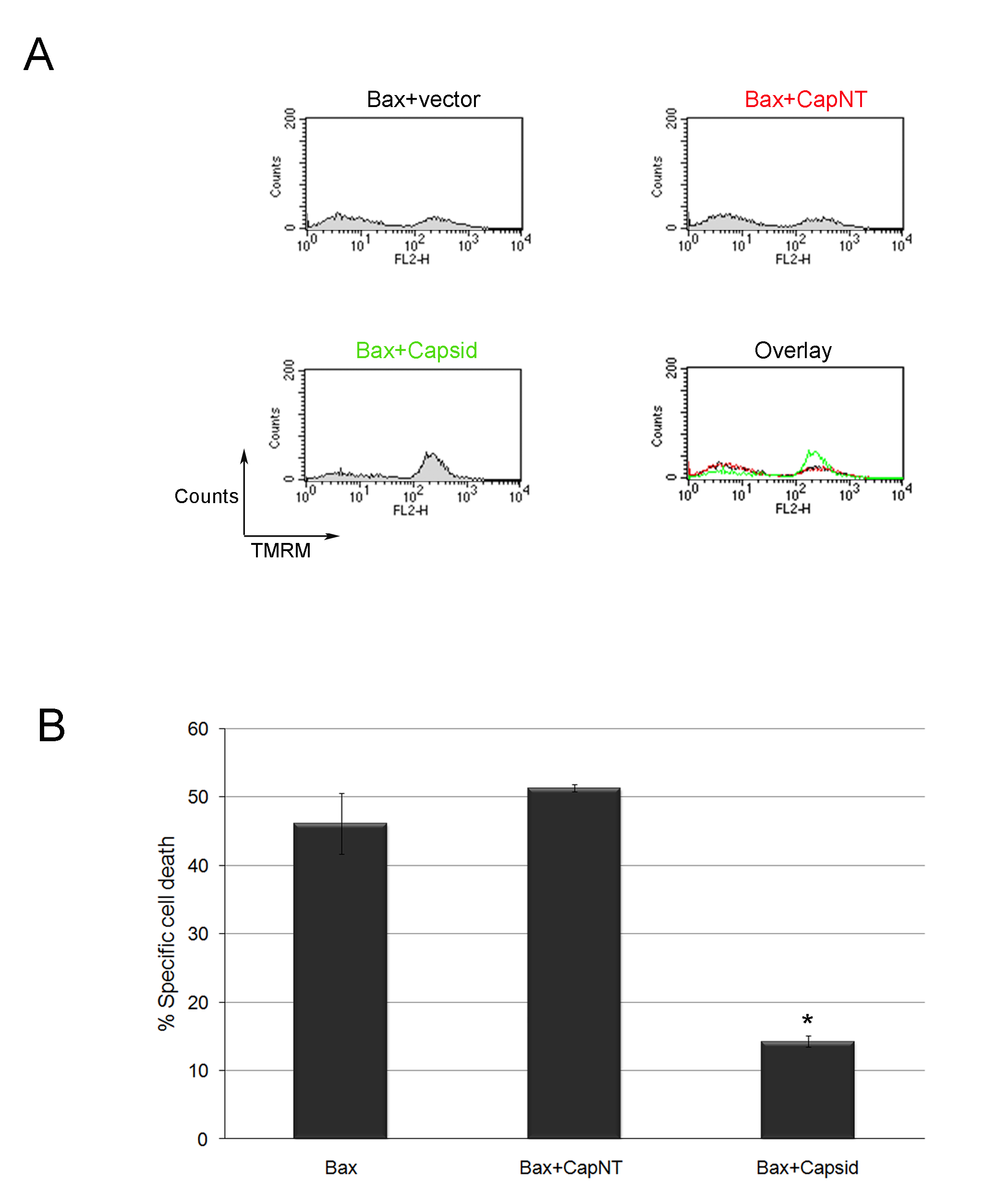

Supplement: Figure S4 — Expression of capsid protein protects primary human cells from Bax-induced apoptosis. HEL-18 cells cells were co-transfected with plasmids encoding GFP-Bax together with plasmids encoding capsid, CapNT, or vector alone. After 24 hours, samples were stained with TMRM for 30 minutes and then subjected to flow cytometric analyses. A. Sample FACS plots for GFP-Bax transfectants are shown. B) The levels of relative specific cell death in 5,000 GFP positive cells were calculated and plotted. Error bars indicate standard deviations calculated from three independent experiments. (0.40 MB TIF) [file ppat.1001291.s004.tif]

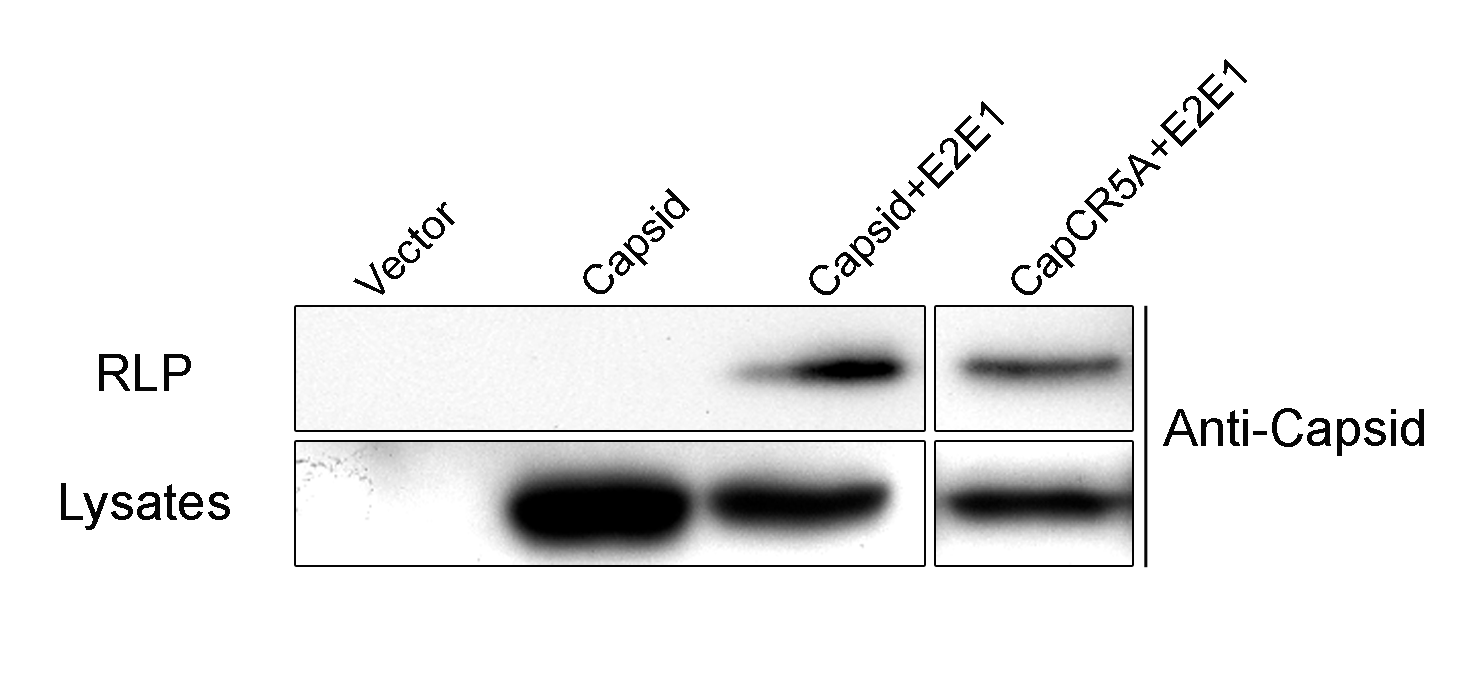

Supplement: Figure S5 — The CapCR5A mutant is fully functional for assembly of rubella virus like-particles (RLP). Vero cells were transfected with plasmids encoding vector; WT capsid alone (Capsid); or a plasmid encoding glycoproteins E2 and E1 with Capsid or CapCR5A. After 48 hours, RLPs recovered from the pre-cleared media by centrifugation at 100,000 x g were detected by immunoblotting with a rabbit polyclonal antibody. (0.18 MB TIF) [file ppat.1001291.s005.tif]
